# Form Meets Function: Fiber Architecture Directs Proliferation and Differentiation in Gingival Keratinocytes

**DOI:** 10.3390/cells15030300

**Published:** 2026-02-05

**Authors:** Imke Ramminger, Thorsten Steinberg, Bernd Rolauffs, Mischa Selig, Pascal Tomakidi

**Affiliations:** 1 Division of Oral Biotechnology, Center for Dental Medicine, Medical Center—University of Freiburg, Faculty of Medicine, University of Freiburg, Hugstetterstr. 55, 79106 Freiburg, Germany; 2 Faculty of Biology, University of Freiburg, Schaenzlestr. 1, 79104 Freiburg, Germany; 3 G.E.R.N. Center for Tissue Replacement, Regeneration & Neogenesis, Department of Orthopedics and Trauma Surgery, Medical Center-Albert-Ludwigs-University of Freiburg, Faculty of Medicine, Albert-Ludwigs-University of Freiburg, Hugstetter Strase 55, 79106 Freiburg, Germany

**Keywords:** scaffold, electrospinning, fiber orientation, fiber diameter, morphology, nuclear shape/morphology, proliferation, differentiation, keratinocyte, gingiva

## Abstract

Precise control of keratinocyte proliferation and differentiation is critical for oral epithelial regeneration, yet the mechanobiological cues guiding these processes remain incompletely defined. Here, we systematically evaluated how electrospun polycaprolactone (PCL) scaffolds with defined fiber orientations (aligned vs. random) and diameters (600–800 nm, 1.2–1.7 µm, 2.0–2.5 µm) direct gingival keratinocyte fate. Using immortalized human gingival keratinocytes, we assessed cell and nuclear morphology, proliferation dynamics, differentiation marker expression, and the effects of basal keratin (KRT5/KRT14) knockdown. Quantitative morphological analysis revealed scaffold-dependent changes in cell shape: aligned medium-diameter fibers (with fiber diameters of 1.2–1.7 µm) induced pronounced cell and nuclear elongation, whereas random fibers (600–800 nm) promoted larger, more rounded cell and nuclear shapes. Time-resolved EdU assays indicated that aligned scaffolds supported sustained proliferation, whereas random scaffolds elicited a transient proliferative burst followed by a decline. Gene expression analysis (ddPCR) demonstrated that random scaffolds (especially 600–800 nm fibers) upregulated basal keratins (KRT5, KRT14) and early differentiation markers (KRT1, KRT10, KRT4, KRT13) relative to aligned scaffolds. At the protein level, differentiation markers involucrin (IVL) and filaggrin (FLG) were likewise elevated on random scaffolds, corroborating the mRNA findings. Functional KRT5/KRT14 knockdown experiments revealed scaffold-specific dependencies: cells on random scaffolds required these keratins for viability, whereas aligned cultures remained viable upon KRT5/14 loss. Furthermore, KRT5/14 depletion differentially altered downstream differentiation markers (IVL, KRT1) and mechanotransduction markers (LMNB1, YAP1) in a scaffold-dependent manner. Collectively, these findings establish fiber orientation and diameter as key design parameters for controlling keratinocyte fate. As a translational concept, layered scaffolds combining aligned and random fibers may enable spatially controlled proliferation and differentiation in engineered oral epithelia.

## 1. Introduction

Regeneration of the oral epithelium following trauma, disease, or surgical intervention requires orchestrated keratinocyte proliferation and differentiation within a biomechanically active microenvironment. Unlike static in vitro models, the in vivo gingival epithelium is continuously exposed to mechanical perturbation, microbial challenges, and biochemical gradients. The clinical demand for reliable epithelial regeneration strategies, particularly in the context of periodontal disease, implant integration, or mucosal reconstruction, has driven the development of biomaterials that recapitulate structural and functional aspects of native epithelial tissues [1,2].

Keratinocytes, the dominant epithelial cell type, depend on an intact cytoskeletal network composed of keratins, actin filaments, and microtubules to maintain mechanical integrity and barrier function. Among these, basal keratins KRT5 and KRT14 are central to providing resilience against deformation while anchoring molecular complexes involved in signaling and adhesion [3,4,5,6]. As keratinocytes differentiate, they downregulate KRT5/14 and upregulate suprabasal markers such as KRT1, KRT10, and involucrin (IVL), reflecting a transition from proliferative to differentiated states [7,8].

Mechanical signals from the extracellular matrix (ECM) contribute to these transitions through changes in substrate stiffness, geometry, and topography. These cues are perceived via integrin-based adhesion complexes and cytoskeletal linkages, influencing cell shape, nuclear architecture, and gene expression programs [9,10]. In epithelial systems, pathways such as YAP/TAZ have been implicated in transducing mechanical input into transcriptional responses, although their precise roles in scaffold-guided keratinocyte fate remain to be clarified [11].

Electrospun polycaprolactone (PCL) scaffolds present a versatile platform to study such mechanobiological processes in vitro. By tuning parameters such as fiber diameter and orientation, these scaffolds mimic structural features of the native ECM while providing defined mechanical and topographical cues [12,13]. Electrospinning (ES) is a widely used technique for fabricating nanofibrous polymer scaffolds, including those made of poly(ε-caprolactone) (PCL) [14]. By applying a high-voltage field to a polymer solution, ES produces continuous fibers with tunable diameters, alignments, and porosities [14]. Key processing parameters, such as the applied voltage, solution feed rate, and collector configuration, can be adjusted to control fiber morphology and mechanical properties. For example, recent work prepared PCL nanofibers via single-needle electrospinning under ~20 kV applied voltage, a 25 cm needle-to-collector distance, and a 0.5 mL/h flow rate, yielding uniform microfiber mats [15]. These represent typical conditions for PCL fiber production [15], underscoring the reproducibility of ES in generating scaffolds for tissue engineering.

While widely applied in mesenchymal contexts, the epithelial response to scaffold geometry, particularly in gingival keratinocytes, remains insufficiently explored. Previous studies have shown that keratinocyte morphology and fate decisions are sensitive to topographical features such as fiber alignment and nanoscale curvature [16,17]. However, mechanistic insight into how these structural parameters translate into changes in proliferation, differentiation, and cytoskeletal organization is lacking. In particular, the role of basal keratins KRT5 and KRT14 in mediating scaffold-specific epithelial responses has not been defined.

Electrospun fibers with controlled alignment and diameter have been used to modulate the behavior of skin-derived cells. Poyraz et al. demonstrated that random versus aligned polycaprolactone (PCL) fibers influence dermal fibroblast and keratinocyte adhesion and morphology [18], while Zhou et al. showed that tilapia–collagen nanofibers accelerated wound healing by enhancing keratinocyte proliferation and differentiation [19]. Fu et al. reported that multiscale collagen nanofiber matrices regulate keratinocyte migratory activity through topographical cues [20]. Pelipenko et al. discussed how fiber diameter critically affects skin cell response, with smaller diameters promoting spreading and differentiation [21]. However, these studies typically examined single cell types or single diameters and did not analyze nuclear morphology or mechanotransduction pathways. Our study advances this field by systematically varying PCL fiber diameter across three ranges (600–800 nm, 1.2–1.7 µm, 2.0–2.5 µm) and changing orientations (aligned vs. random) to modulate keratinocyte fate. Using immortalized human gingival keratinocytes (ihGK), we assess changes in cell and nuclear morphology, proliferation dynamics over time, expression of early and late differentiation markers (KRT1, KRT10, IVL), and the functional relevance of KRT5/KRT14 via siRNA-mediated depletion. These readouts provide both structural and transcriptional insight into how scaffold architecture governs epithelial fate.

We hypothesize that fiber diameter and orientation act as tunable biomechanical signals modulating keratinocyte fate via KRT5/KRT14-dependent pathways. Based on previous work in epithelial mechanobiology, we propose that submicron random fibers may promote differentiation-associated programs, whereas aligned architectures could favor basal phenotype preservation and sustained proliferation. This study is designed to characterize how distinct scaffold architectures shape proliferation dynamics and the expression of markers associated with basal and suprabasal differentiation states in gingival keratinocytes.

## 2. Materials and Methods

### 2.1. ihGK Cell Cultivation on Polycaprolactone Nanofiber Scaffolds

Primary human gingival keratinocytes were isolated from healthy gingival tissue with written informed consent of the donor and approval by the Ethics Committee of Heidelberg University (Approval ID: 148/2003, date 30 September 2005) and in accordance with the principles outlined in the Declaration of Helsinki (1975, revised in 2013). These primary cells were immortalized with the E6/E7 gene of the human papilloma-virus type 16, while retaining all biomarkers of native gingival epithelial tissue including keratins of interest as well as IVL and FLG [22]. The cells were cultured in passages 38–49 in Keratinocyte growth medium supplemented with supplement mix, CaCl_2_ (0.06 mM) (KGM-2, Promocell, Heidelberg Germany) and kanamycin (100 µg mL^−1^) (Sigma-Aldrich, St. Louis, MO, USA). Cultures were kept at 37 °C in a humidified 5% CO_2_ incubator, and the medium was replaced every 3–4 days. These standard conditions ensured robust cell viability throughout the 3- or 7-day culture period on both control and PCL scaffold substrates.

All polycaprolactone (PCL) nanofiber scaffolds used in this study were obtained from a commercial supplier (The Electrospinning Company Ltd., Didcot, UK) in two different configurations, aligned and random, and three different fiber diameters, 600–800 nm, 1.2–1.7 µm and 2.0–2.5 µm, rather than produced in-house. Consequently, the precise electrospinning process parameters (e.g., polymer solution concentration, applied voltage, and flow rate) employed to generate these scaffolds are proprietary to the manufacturer and were not variables under investigation in this work. Nevertheless, key material descriptors of the scaffolds were independently verified to ensure they met the intended specifications. Scanning electron microscopy confirmed the expected fiber architectures (highly aligned fibers vs. randomly oriented fibers), and quantitative image analysis yielded mean fiber diameters that fell within the nominal ranges for each scaffold type (as reported in Supplementary Table S1). For example, the aligned 600–800 nm fiber scaffolds exhibited an average fiber diameter of 926 ± 366 nm, consistent with the supplier’s target range (Supplementary Table S1). The three fiber diameter ranges used in this study were deliberately chosen to span the transition from nanoscale to microscale fibers and to probe potential non-linear effects on cell behavior. Fibers in the 600–800 nm range approximate the diameter of native collagen fibrils in the extracellular matrix, whereas 1.2–1.7 µm and 2.0–2.5 µm fibers extend into the multi-micron regime. Previous reports have shown that keratinocytes exhibit distinct responses at different fiber sizes, with intermediate diameters sometimes producing optimal proliferation and differentiation. Including an intermediate group (~1.5 µm) thus allowed us to determine whether gingival keratinocytes display progressive or threshold-type responses to fiber dimension [21,23]. Additional reasons for using the three different fiber diameters were to span physiologically relevant length scales. Sub-micrometer fibers (~600–800 nm) mimic the curvature of the basement membrane and have been shown to enhance cell spreading and differentiation compared with larger fibers [24]. Medium-sized fibers (1.2–1.7 µm) provide a moderate curvature that can sustain proliferation and alignment. Larger fibers (2.0–2.5 µm) reduce curvature and approach planar surfaces, allowing us to determine whether gingival keratinocytes respond differently when the topographical cue becomes less pronounced. Previous studies on myoblasts and stem cells found that aligned fibers ranging from ~335 nm to several micrometers facilitate alignment and differentiation, with larger diameters often promoting enhanced differentiation [25]. By including all three ranges, our design tests whether keratinocyte behavior is sensitive to subtle changes in fiber diameter.

Although the scaffolds were sourced commercially, the manufacturer tuned standard electrospinning parameters (PCL concentration, applied voltage and flow rate) to achieve the specified diameter ranges. In general, lower polymer concentration and moderate voltage yield submicron fibers, whereas higher concentration or adjusted voltages produce thicker microfibers. These typical parameter ranges (e.g., 10–15% *w*/*v* PCL in a fluoro-alcohol solvent at 10–20 kV) are consistent with published PCL electrospinning protocols [26]. The electrospun PCL mats used in this study were thin, pliable sheets on the order of tens of micrometers in thickness. Based on supplier specifications and our observations, the random fiber mats were approximately 50 µm thick, whereas the aligned fiber mats were slightly thinner. This ~50 µm scaffold depth is consistent with commercially available electrospun membranes that provide a 3D architecture yet remain thin enough for nutrient diffusion and imaging. All scaffolds in our batches fell below roughly 50 µm in thickness, in line with the manufacturer’s data (Electrospinning Company, personal communication).

Two batches of electrospun PCL scaffolds were used in this study. Batch 1 (initial lot) comprised both aligned and random fiber scaffolds in three nominal fiber diameter ranges: 600–800 nm, 1.2–1.7 µm, and 2.0–2.5 µm. Batch 2 (subsequent lot) comprised aligned and random fiber scaffolds in two diameter ranges (600–800 nm and 1.2–1.7 µm); the largest fiber diameter group (2.0–2.5 µm) was not included in batch 2, since initial findings from batch 1 indicated that the 2.0–2.5 µm fibers had the least pronounced effects on gingival keratinocyte behavior compared to the smaller fiber scaffolds. Both batches were obtained from the same manufacturer under the same fabrication parameters to ensure comparable fiber characteristics.

The electrospun scaffolds were obtained in two distinct batches. Batch 1 was used primarily for initial scaffold characterization, imaging, cell morphology measurements, and preliminary gene expression profiling (via ddPCR) at 3 and 7 days. By the end of these initial experiments, the batch 1 scaffolds were fully utilized. Consequently, all subsequent functional assays, including EdU proliferation analysis, fluorescence intensity quantification of protein markers, siRNA knockdown experiments, and the associated qPCR gene expression analyses, were carried out using batch 2 scaffolds. In the second batch, note that scaffolds in the largest fiber diameter range (2.0–2.5 µm) were not available, but the small (600–800 nm) and medium (1.2–1.7 µm) fiber diameter scaffolds in batch 2 produced qualitatively similar cell responses as observed with batch 1. Performing the functional studies on batch 2 was necessary because no batch 1 scaffolds remained for further experimentation. Importantly, the topography-driven trends in cell behavior were consistent between batch 1 and batch 2, ensuring that the findings from the batch 2 functional assays are comparable and representative of the effects observed in batch 1.

Electrospun scaffold morphology was characterized by scanning electron microscopy (SEM). Scaffolds without cells were mounted onto stubs, sputter-coated with a thin gold layer, and imaged using an SEM (ZEISS Evo, Jena, Germany or similar instrument) at various magnifications. Representative micrographs were acquired for each fiber orientation (aligned, random) and nominal diameter range (600–800 nm, 1.2–1.7 µm, 2.0–2.5 µm) from each production batch. To quantify fiber diameters, the SEM images were analyzed using ImageJ, version 1.54i (NIH, Bethesda, MA, USA). The diameters of ≥50 fibers per scaffold were measured to calculate the mean fiber diameter and standard deviation for each scaffold type. These values, grouped by fiber orientation, diameter range, and batch, are reported in Supplementary Table S1.

For the cultivation of ihGKs on the different PCL nanofiber scaffolds, the scaffolds were first carefully inserted into a 12-well plate. For the control, cells were seeded directly into the plate (RNA isolation) or a pre-sterilized 12 mm coverslip (for staining) was inserted into the plate. Materials were washed with 70% ethanol in sterile ddH_2_O for 10 min on a vertical rocker to promote cell attachment. The materials were washed three times for 5 min at RT with sterile ddH_2_O on a vertical rocker, followed by a 30 min incubation step with 500 µL of fresh full KGM in the incubator in a humidified atmosphere at 37 °C and 5% CO_2_. Next, KGM was replaced with 500 µL fresh full KGM. Cells were seeded at 8 × 10^3^ cells/well or 2 × 10^4^ cells/well in 1 mL KGM each, depending on experimental conditions and cultured for 3 or 7 days. For the seven-day cultivation period, KGM was replaced with 1.5 mL of fresh full prewarmed KGM after 3 days. Two seeding densities were used based on the specific assay requirements. For morphological analyses and proliferation assays, cells were seeded at 8 × 10^3^ cells per well to avoid confluence by day 7. For siRNA knockdown experiments, a higher density of 2 × 10^4^ cells per well was necessary to achieve efficient transfection and sufficient RNA/protein yield. Transfection efficiency depends on cell confluency. Commercial guidelines recommend plating cells to reach 40–80% confluence at the time of transfection and note that too few or too many cells per well can reduce siRNA delivery. Our empirically determined density ensured that keratinocytes were approximately 50% confluent at the time of transfection, maximizing knockdown while maintaining cell viability.

### 2.2. Droplet Digital PCR (ddPCR) (Batch 1)

For the differentiation analysis with ddPCR ihGK cells were seeded at 8 × 10^3^ cells/well and cultivated for 3 or 7 days. RNA was isolated with the RNeasy Micro Plus Kit (Qiagen, Hilden, Germany) under the use of QIAshredder tubes (Qiagen). RNA was quality-checked using the Experion RNA HighSens Analysis system (Bio-Rad, Hercules, CA, USA), and 50–200 nm RNA was converted to cDNA with the Advantage RT-for-PCR Kit (Takara, Kusatsu, Japan) using 1 µL of random hexamer primers and 1 µL of oligo(dT)18 primer, both according to manufacturer’s protocol. For ddPCR, 3 ng cDNA was used per reaction, except for KRT5 and KRT14 with 1 ng cDNA input, to quantify two target genes simultaneously. Droplets (~20,000/sample) were generated using the Bio-Rad AutoDG droplet generator and transferred to a 96-well plate for thermal cycling on a QX100 system. Fluorescence signals were analyzed with the QX200 droplet reader, and gene expression was quantified using QuantaSoft (v1.7.4). A two-color detection system (FAM/HEX) enabled multiplexing in a single well. Each 22 µL reaction included 15.4 µL ddPCR Supermix for Probes (no dUTP), 1.1 µL each of 20× PrimePCR primer/probe mixes (FAM and HEX), 2.2 µL nuclease-free water, and 6.6 µL diluted cDNA. All Primers were purchased from Bio-Rad (KRT1, FAM AssayID: dHsaCPE5049864; KRT4, FAM AssayID: dHsaCPE5057768, KRT5, FAM, AssayID: dHsaCPE5042494, KRT10, HEX, AssayID: dHsaCPE5042547, KRT13, HEX, AssayID: dHsaCPE5046071, KRT14, HEX, AssayID: dHsaCPE5192232, IVL, FAM, AssayID: dHsaCPE5039360, FLG, HEX, AssayID: dHsaCPE5029907). PCR cycling conditions were as follows: 95 °C for 10 min; 40 cycles of 94 °C for 30 s and 55 °C for 1 min; 98 °C for 10 min; hold at 4 °C. Droplets were classified as positive based on fluorescence intensity, with primer-specific thresholds maintained across biological replicates. No technical replicates were performed due to limited RNA availability.

### 2.3. Morphology Analysis of ihGK Cells (Batch 1 and Batch 2)

The morphology analysis of ihGK cells and their nuclear shape was performed with an ImageJ/Fiji analysis algorithm, which measured the shape descriptors of cell area (µm^2^), aspect ratio (ratio of major to the minor axis of the cell), roundness (4 × area/(π × major axis^2^)), circularity (4*π(area/perimeter^2^)) and solidity (area/convex area (cell)). Therefore, the parameters of aspect ratio and roundness describe the cell elongation while the parameters of circularity and solidity describe cell protrusions/indentions. The analysis algorithms were obtained from Dr. Mischa Selig from the research group of Prof. Dr. Bernd Rolauffs [27,28]. Imaging of the first and second scaffold batch was conducted with different microscopes. Furthermore, the analysis algorithm was first designed to detect mesenchymal stem cells and therefore, the algorithm was adapted to fit ihGK cell requirements. ihGK cells are smaller in cell size and tend to accumulate in groups. In the second scaffold batch, in particular, ihGK cells were clustered. Hence, a separation function was included in the algorithm to separate the cells. Generally, the algorithm was designed to detect a single cell or a single nucleus to measure the mentioned shape descriptor. As not all cells/nuclei were detected correctly, a manual query was integrated to decide between correctly and wrongly detected cells. For cell/nucleus detection, ihGK cells were seeded at 8 × 10^3^ cells/well, cultivated for 3 or 7 days and stained for actin (Phalloidin 488, Thermo Fischer Scientific, Waltham, MA, USA) and nuclei (DAPI, Thermo Fischer Scientific, Waltham, MA, USA) (staining protocol in detail with PFA fixation in Section 2.5). For the first experimental batch, fluorescence microscopy was performed using an Axio Observer Z1 inverted microscope (Zeiss, Oberkochen, Germany). For the second batch, images were acquired with a BZ-9000 digital fluorescence microscope (Keyence, Neu-Isenburg, Germany). All images were captured using a 20× objective lens, and identical calibration was applied to ensure a consistent scale in micrometers. In both batches, imaging conditions (exposure time, filter sets, and image acquisition settings) were kept constant across all samples to allow unbiased comparison. Furthermore, the same ImageJ/Fiji analysis pipeline was used for quantitative cell morphology measurements in batch 1 and batch 2, ensuring that data from both imaging setups could be directly compared.

### 2.4. Proliferation Analysis with Click-iT EdU (Batch 2)

The proliferation activity was measured with the Click iT EdU Cell proliferation Kit from Thermo Fischer Scientific (Waltham, MA, USA) according to the manufacturer’s protocol. ihGK cells were seeded at 8 × 10^3^ cells/well for 3 and 7 days. EdU incubation was set to 6 h. Imaging was performed with a Keyence BZ-9000 fluorescence microscope (Neu-Isenburg, Germany) and EdU positive cells were counted and compared to whole nuclei counts.

### 2.5. Indirect Immunofluorescence Staining and Protein Intensity Measurement (Batch 2)

ihGK cells (8 × 10^3^/well) were cultivated for 3 and 7 days on the different surfaces. After the incubation period, cells were washed thrice with warm 1× Dulbecco’s phosphate-buffered saline (DPBS) (Thermo Fischer Scientific, Waltham, MA, USA) and fixed for 20 min at RT using warm 4% Paraformaldehyde (PFA, Carl Roth, Karlsruhe Germany) for anti-KRT5, -KRT14, -KRT1, -IVL staining or with ice-cold 100% ethanol (VWR International, Radnor, PA, USA) for 10 min for anti-KRT4 staining. After three rinses with DPBS, cells were permeabilized with 0.1% Triton-X-100 (Sigma-Aldrich, St. Louis, MO, USA) in DPBS for 15 min at RT, washed with DPBS three times and incubated in 2% BSA in DPBS (Sigma-Aldrich, Burlington, MA, USA) for 1 h at RT. Afterwards, cells were directly incubated with the primary antibodies in 0.5% BSA in DPBS overnight at 4 °C (volumes: 45 µL for coverslips, 300 µL for random, 600 µL for aligned scaffolds; dilutions: anti-KRT5, Abcam, Cambridge GB, #ab52635, 1:250; anti-KRT14, Thermo Fischer Scientific, Waltham, MA, USA, #MA5-11599, 1:250; anti-KRT1, Abcam, #ab93652, 1:200; anti-IVL, Abcam, #ab53112 1:100; anti-KRT4, Abcam, #ab92465, 1:250). After washing thrice with DPBS, secondary antibodies (goat-anti-mouse 488, Thermo Fischer Scientific, Waltham, MA, USA, 1:200 or goat-anti rabbit 488, Thermo Fischer Scientific, 1:200) and Phalloidin (Phalloidin 594, Abcam, Cambridge, MA, USA, 1:1000) were applied for 1 h at RT using matching volumes. Samples were then washed and counterstained with DAPI (300 nM, Thermo Fischer Scientific, Waltham, MA, USA) for 15 min at RT. After final washes, samples were mounted in ProLong Glass Antifade Mounting medium (Thermo Fischer Scientific, Waltham, MA, USA). Coverslips were mounted cell-side down; scaffolds were mounted cell-side up with a 12 mm coverslip on top. Imaging was performed using a Keyence BZ-9000 fluorescence microscope. For imaging, the exposure time was set the same between samples with the same protein staining. Protein intensity was analyzed using the same Fiji/ImageJ algorithm applied for morphology, extended to quantify fluorescence levels of batch 2 cells. This algorithm further included the pixel intensity measurement of the protein of interest. The fold change of the mean protein intensity value of one sample was calculated to the mean protein intensity value of the control for each biological replicate.

### 2.6. Scanning Electron Microscopy (SEM)

Samples with cells (8 × 10^3^/well) were fixed with 4% PFA in DPBS for 20 min at RT after 3 or 7 days of incubation. Samples were rinsed three times with DPBS and dehydrated through increasing ethanol concentrations (30%, 50%, 70%, 80%, 90%, 100%). Critical point drying (CPD) was performed using liquid CO_2_ with CPD 030 critical point dryer (BAL-TEC, Balzers, Liechtenstein). Due to scaffold fragility, some samples were partially dried or damaged during this process. Dried samples were sputter-coated with gold for 30–40 s (JFC-1200 Fine Coater, Jeol, Akishima, Japan); cell-free samples were coated without CPD. Samples were stored dry and imaged using a JSM-IT100 Microscope (Jeol) at various magnifications.

### 2.7. Knock-Down with siRNA (Batch 2)

Cells were seeded at 2 × 10^4^ cells/well for 24 h on the different surfaces, prior to siRNA-mediated knock-down of KRT5 and KRT14, with non-targeting siRNA for control (50 pmol, Silencer Select siRNAs, Life Technologies GmbH, Darmstadt, Germany) according to manufacturer’s protocol (siRNA KRT14, #4392420; siRNA KRT5, #4392421 and nt-siRNA, #4390844, Thermo Fischer Scientific, Waltham, MA, USA). The knock-down was performed for 72 h, followed by RNA isolation with the RNeasy Plus Micro Kit (Qiagen, Hilden, Germany) under the use of QIAshredder tubes (Qiagen). The commercially available siRNAs were selected in such a way that an inhibitory effect of at least 80% was achieved on mRNA levels.

### 2.8. qPCR (Batch 2)

The isolated mRNA from siRNA-treated cells were measured for concentration and integrity with the 4150 TapeStation system (Aqilent, SantaClara, CA, USA) according to the manufacturer’s protocol. Afterwards, cDNA synthesis was performed with the Advantage RT-for-PCR kit (Takara, Kusatsu, Japan) according to the manufacturer’s protocol and up to 300 ng RNA was synthesized to cDNA. 1 µL of random hexamer primers and 1 µL of oligo(dT)18 primer were used per sample. For qPCR analysis, 8 ng of cDNA was used to quantify the gene of interest in duplicate technical experiments. cDNA was mixed with a SYBR Green reaction cocktail (Qiagen, Hilden, Germany). All primers were purchased from Qiagen (ITGB1 # PPH00650B, ITGB3 #PPH00178D, IVL #PPH01911A, KRT1 #PPH06951F, KRT4 #PPH05703B, KRT14 #PPH02389A, KRT5 #PPH02625F, LMNB1 #PPH00278B, PTK2 (FAK) #PPH02827A, YAP1 #PPH13459A, ACTB #PPH00073G, RPL13A #PPH01020B, UBC #PPH00223F). The analysis of the ΔΔC_t_ value was performed according to Livak and Schmittgen 2001 [29]. ATCB, UBC and RPL13 were used as housekeeping genes.

### 2.9. Statistical Analysis

Data analysis was performed using Microsoft Excel 2016 and GraphPad Prism (Version 7.05). All experiments included 3–4 biological replicates. Data normality was evaluated using the Shapiro–Wilk test. Parametric data were analyzed with an unpaired two-tailed *t*-test, while non-parametric data were assessed using the Mann–Whitney test. Comparisons were made between experimental groups and the control, as well as among fiber orientation and diameter variations. Statistical significance was defined as *p* < 0.05 (* *p* < 0.05, ** *p* < 0.01, *** *p* < 0.001, **** *p* < 0.0001).

## 3. Results

We systematically examined how electrospun PCL scaffolds with defined fiber diameters and orientations affect key biological functions of ihGK. To capture early responses to scaffold topography, we first analyzed changes in cell morphology and nuclear shape across the different scaffold types.

### 3.1. Scaffold Architecture Modulates Early Adhesion and Morphological Polarization in ihGK Cells

**Figure 1:**
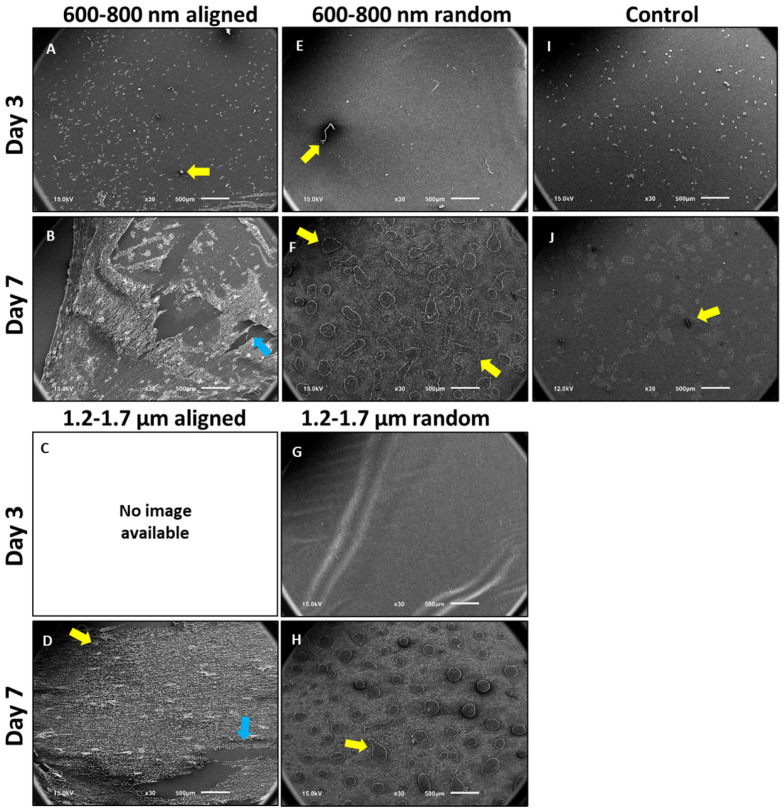
SEM images showing ihGK cell adhesion and morphology on electrospun scaffolds with different fiber architectures after 3 and 7 days of culture. (**A**–**D**) Aligned scaffolds with fiber diameters of 600-800 nm (**A**,**B**) and 1.2–1.7 µm (**C**,**D**); (**E**–**H**) random scaffolds with fiber diameters of 600–800 nm (**E**,**F**) and 1.2–1.7 µm (**G**,**H**); (**I**,**J**) planar control surfaces. All images were captured at low magnification (×30) to visualize cellular distribution. Aligned 600–800 nm scaffolds supported progressive ihGK cell attachment and surface coverage, especially evident by day 7 (**B**). A similar trend was noted on aligned 1.2–1.7 µm scaffolds, though partial scaffold rupture occurred during sample preparation (indicated by blue arrows in (**B**,**D**)). Yellow arrows highlight representative artifacts, such as CPD-induced damage or debris. Overall, SEM imaging demonstrates that scaffold topography strongly influences ihGK adhesion and early morphological behavior. Scale bar: 500 μm, *n* = 1. [30]. Corresponding SEM micrographs of the unseeded fiber scaffolds (illustrating fiber orientation and diameter) are provided in Figure S19, and the fiber diameter measurements for each scaffold type (batch 1 vs. batch 2) are summarized in Table S1.

**Figure 2:**
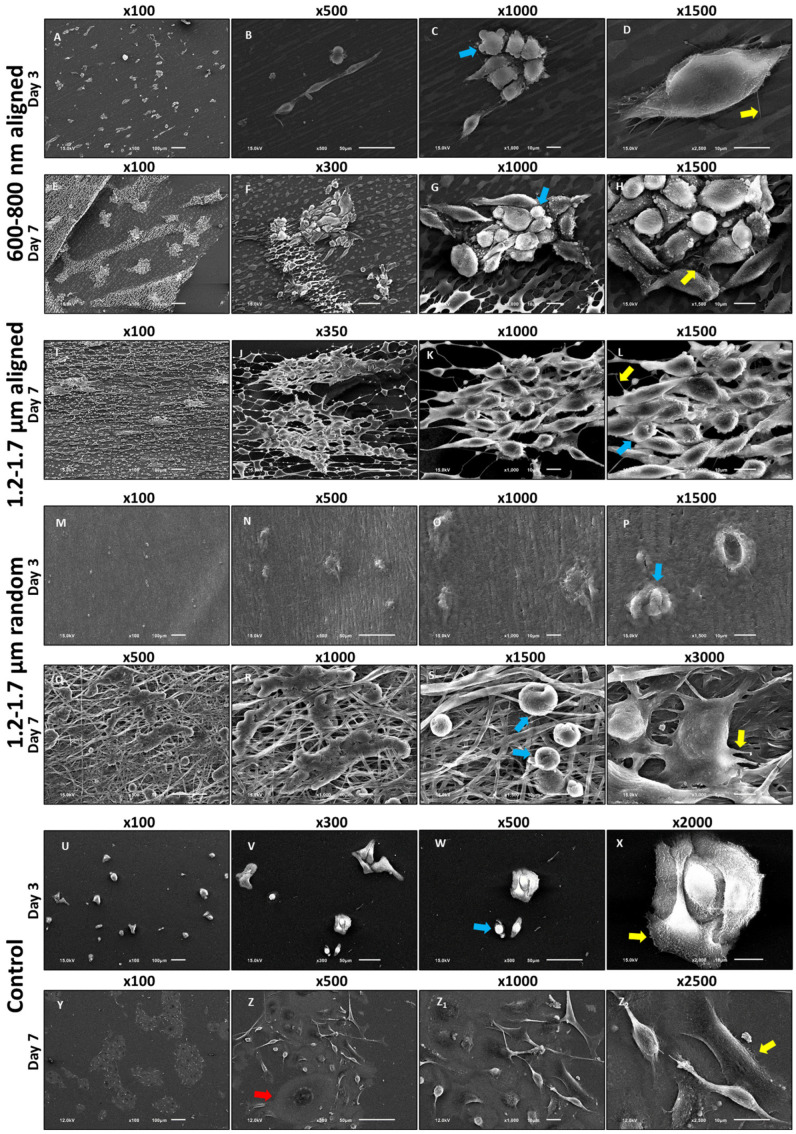
Scanning electron microscopy (SEM) images of immortalized human gingival keratinocytes (ihGK) cultured on different surfaces for 3 and 7 days. Cells were fixed, dehydrated by critical point drying and sputter-coated with a thin gold layer prior to imaging. Panels show representative micrographs of ihGK on aligned scaffolds (**A**–**L**), randomly oriented scaffolds (**M**–**X**), and flat control surfaces (**Y**–**Z_2_**). Images were acquired at multiple magnifications to visualize cell attachment, morphology and scaffold architecture. Yellow arrows mark adhesion points and cell protrusions; blue arrows highlight blebbing or a rounded and compact morphology; the red arrow indicates an unusually large cell. Scale bars: 100 µm (100×), 50 µm (300–500×), 10 µm (1000–2500×) and 5 µm (3000×). Representative images are shown from one experiment (*n* = 1) [30].

To test whether scaffold architecture modulates epithelial cell adhesion and early morphological responses, we performed scanning electron microscopy (SEM) after 3 and 7 days of ihGK culture on electrospun scaffolds with defined fiber alignment and diameter. SEM revealed pronounced topography-dependent differences in ihGK cell morphology and distribution. Cells were cultured for 3 and 7 days on aligned (Figure 1A–D) and random (Figure 1E–H) electrospun scaffolds (600–800 nm and 1.2–1.7 µm), as well as planar controls (Figure 1 and Figure 2). Scaffolds with largest fiber diameter (2.0–2.5 µm) were not included in the second scaffold batch. Consequently, no cell-seeded 2.0–2.5 µm samples were available for imaging in Figure 1 and Figure 2.

At low magnification (×30), aligned 600–800 nm (Figure 1A,B) scaffolds supported increasing cell density over time, with robust surface coverage by day 7 (Figure 1B). A similar pattern was observed on aligned 1.2–1.7 µm scaffolds (Figure 1D), despite partial scaffold rupture during sample preparation (Figure 1B,D, blue arrows). In contrast, random scaffolds, irrespective of fiber diameter, showed sparse cell adhesion at both time points (Figure 1E–H). Planar controls (Figure 1I,J) displayed evenly distributed cells that formed compact epithelial islands by day 7 (Figure 1J).

High-resolution SEM confirmed these patterns. On aligned scaffolds, ihGK cells adopted an elongated shape with prominent membrane protrusions at their termini, suggestive of focal adhesion sites at the cell–fiber interface (Figure 2, yellow arrows). These filopodia-like extensions likely represent integrin-rich adhesion complexes by which cells anchor to the fibers [31]. Rounded morphologies and membrane blebbing were also observed in subpopulations (blue arrows). In contrast, random scaffolds predominantly yielded spherical, poorly attached cells. Planar controls exhibited a mix of well-spread and rounded cells (Figure 2Z, red arrow) and larger epithelial clusters.

Sample preparation via critical point drying (CPD) introduced artifacts such as scaffold rupture and surface debris (yellow and blue arrows, Figure 1A–J). Due to single-replicate imaging per condition (*n* = 1), these observations are qualitative.

Together, these data demonstrate that aligned scaffolds promote epithelial elongation and surface retention, whereas random fibers supported a rounded cell shape, similar to control cells. These observations prompted subsequent quantitative morphological analyses.

### 3.2. Fiber Topography Orchestrates Temporal Morphological Adaptation in Keratinocytes

To test whether scaffold topography induces time-dependent morphological changes, ihGK cells were cultured for 3 and 7 days on nanofiber scaffolds with defined fiber orientation (aligned vs. random) and diameter (600–800 nm, 1.2–1.7 µm, 2.0–2.5 µm). Morphometric descriptors, including cell area, aspect ratio (AR), roundness, circularity, and solidity, were extracted from fluorescence images using a semi-automated Fiji/ImageJ workflow (Supplementary Material, Figures S1–S3).

After 3 days, ihGK cells on planar controls exhibited on both batches the largest cell area (Table 1; Figures S4 and S5). Scaffold-grown cells appeared smaller in area than controls, with particularly compact cell sizes on the 600–800 nm fibers (notably on aligned fibers in batch 2). Elongated morphologies were most prominent on aligned scaffolds with 1.2–1.7 µm fibers, reflected by higher AR and lower roundness values (Table 1, Figures S4 and S5). Cells on random scaffolds and on the control remained relatively round, with mean AR values below 2 (close to an un-elongated circular shape).

Circularity and solidity measures were lower for cells on aligned scaffolds in batch 1 (e.g., circularity ~0.30–0.34, solidity ~0.79–0.80) compared to cells on random fibers, consistent with protrusion-rich, elongated cell shapes. (Table 1, Figure S4). Morphometric descriptors such as cell area and perimeter are widely used to assess cell spreading and adhesion on biomaterials [32]. In epithelial tissues, increases in cell area correlate with entry into S phase and cell-cycle progression [33]. We therefore interpret changes in area and perimeter as indicators of proliferation versus differentiation, while acknowledging that different cell types may exhibit distinct morphological responses.

In batch 2, control cells had the highest circularity and solidity, whereas cells on scaffolds had reduced circularity and solidity values indicating more cell protrusions (Table 1, Figure S5). In summary, while batch-specific differences in absolute shape descriptor values were observed, the topography-driven trends remained consistent: in both batches, cells on aligned fiber scaffolds were more elongated, whereas those on random fibers or planar controls remained more compact and rounded. Notably, aligned scaffolds with 1.2–1.7 µm fibers consistently supported the most elongated cell shapes across both batches. These consistent scaffold-specific effects underscore the robust topographical control of epithelial cell shape. (Table 1, Figures S4 and S5).

Morphological differences persisted through 7 days of culture, with aligned fibers promoting an elongated cell shape in both experimental batches (Table 2; Figures S6 and S7). In Batch 1, cells on aligned scaffolds exhibited markedly elongated morphologies—their AR remained elevated (~2.7–3.3) and roundness decreased (~0.46) relative to cells on random fibers or flat controls (AR ~1.8–1.9; roundness ~0.59–0.62, Table 2). This indicates that aligned fiber orientation induces highly elongated cell bodies. Conversely, random fiber scaffolds supported larger, more spread-out cells with relatively round outlines. Cells on random fibers in Batch 1 showed high circularity (~0.34–0.41) and high solidity (~0.80), reflecting smooth, protrusion-poor cell outlines similar to control surfaces. A qualitatively similar trend was observed in Batch 2, although the magnitude of change was attenuated. For example, in Batch 2, the AR on aligned fibers reached ~2.1 (versus ~1.8 on random and control), with a modest decrease in roundness (aligned ~0.54 vs. ~0.57–0.62 on random/control). This batch-specific variability in absolute values underscores that direct comparisons between batches are not appropriate; instead, each batch was evaluated independently to identify consistent scaffold-induced trends in cell shape.

**Figure 3:**
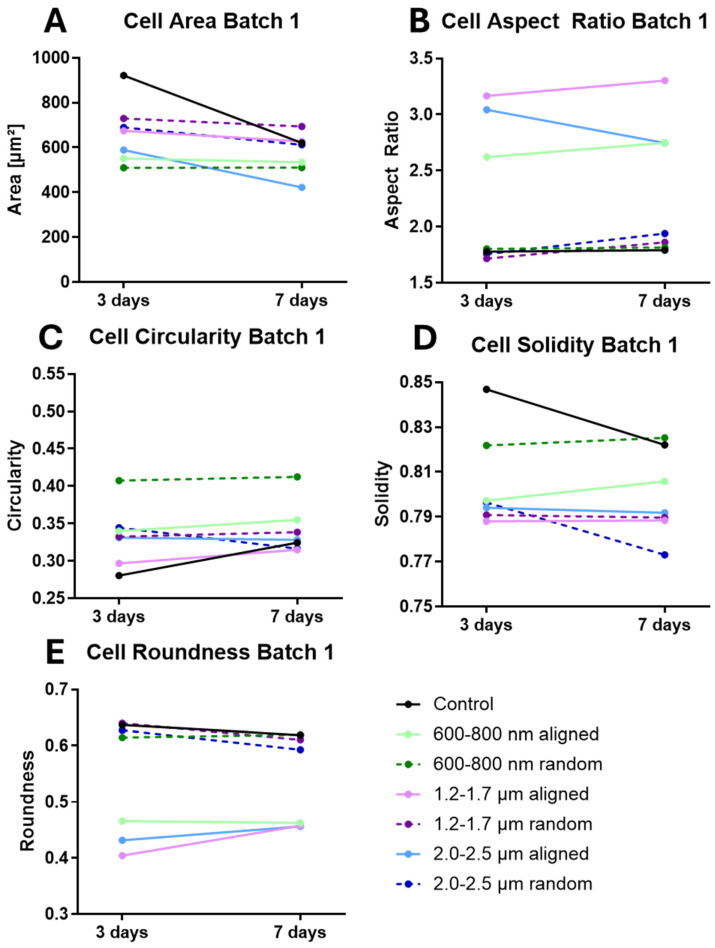
Temporal evolution of mean ihGK cell shape descriptors on various scaffold types at day 3 and day 7 for batch 1. Mean values of ihGK cell morphology metrics measured on different surface topographies. Parameters include (**A**) cell area (µm^2^), (**B**) aspect ratio, (**C**) circularity, (**D**) solidity, and (**E**) roundness. Values represent means calculated from all biological replicates (batch 1: *n* = 4). The corresponding standard deviations (SDs) are provided in Table 1 (for day 3) and Table 2 (for day 7) [30].

**Figure 4:**
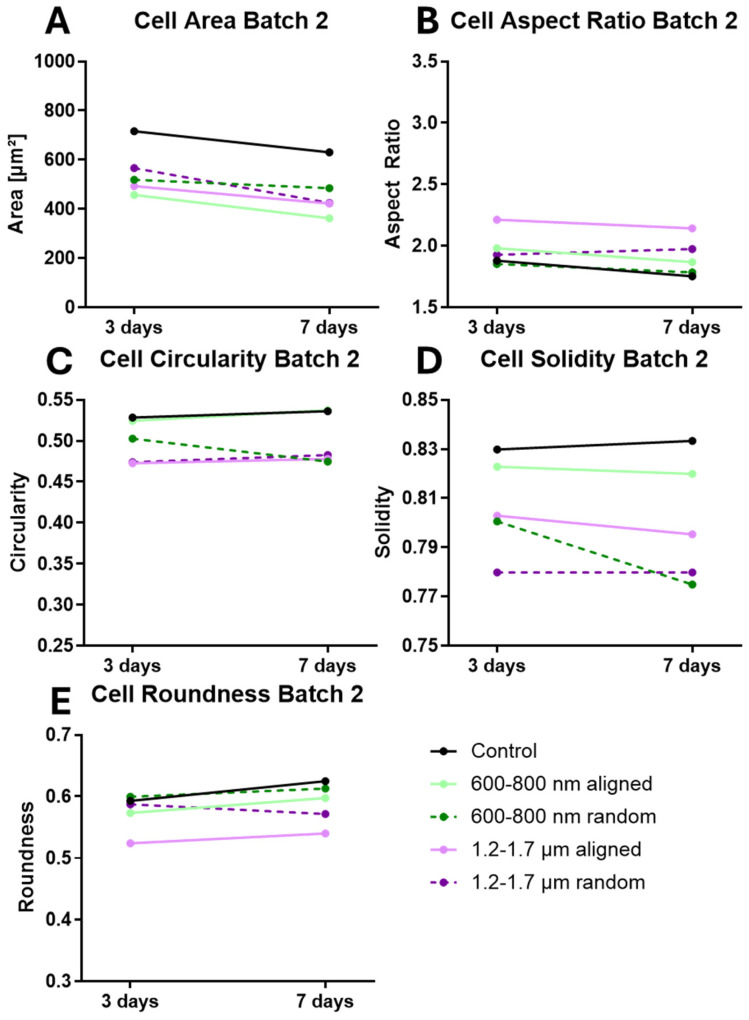
Temporal evolution of mean ihGK cell shape descriptors on various scaffold types at day 3 and day 7 for batch 2. Mean values of ihGK cell morphology metrics measured on different surface topographies. Parameters include (**A**) cell area (µm^2^), (**B**) aspect ratio, (**C**) circularity, (**D**) solidity, and (**E**) roundness. Values represent means calculated from all biological replicates (batch 2: *n* = 3). The corresponding standard deviations (SDs) are provided in Table 1 (for day 3) and Table 2 (for day 7) [30].

Time-resolved analyses confirmed the robustness of these trends in each batch (Figure 3, batch 1 and Figure 4, batch 2). In batch 1, differences in cell shape metrics between aligned and random scaffolds were evident as early as day 3 and became even more pronounced by day 7. Batch 2 showed the same direction of changes over time (aligned fibers driving higher AR and lower roundness than random fibers), but with smaller effect sizes at each time point (Figure 4B,E).

**Figure 5:**
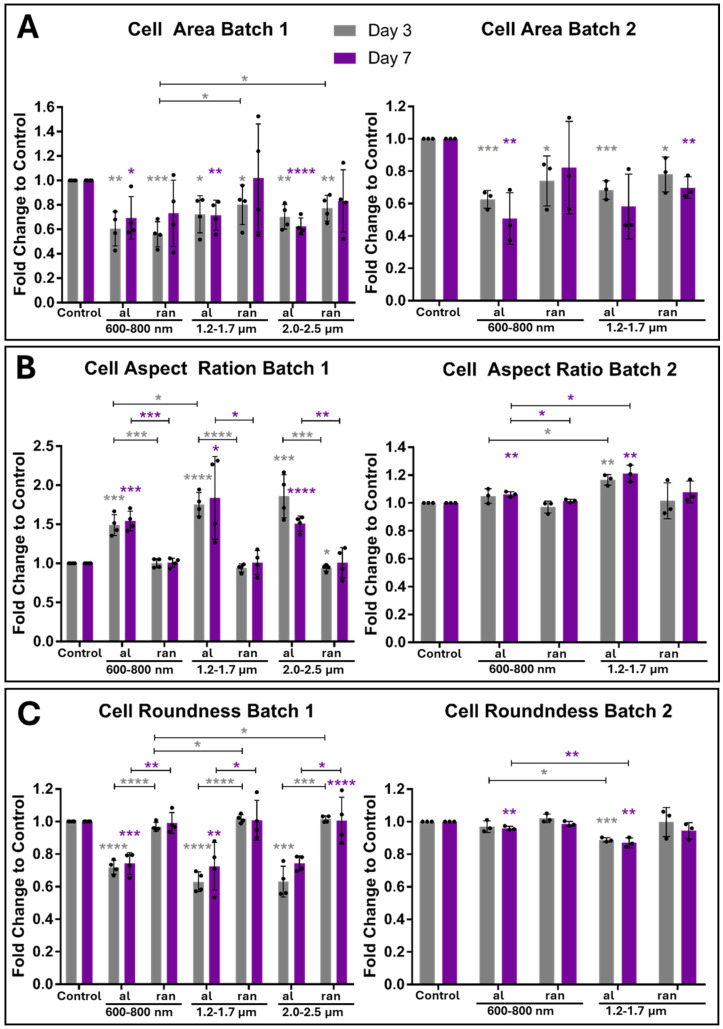
Temporal changes in ihGK cell morphology on various scaffold types compared to control cells at day 3 and day 7. Bar graphs illustrate the fold change in mean values of ihGK cell shape descriptors on different nanotopographic scaffolds, normalized to flat control surfaces, at day 3 and day 7. Quantified parameters include the following: (**A**) cell area, (**B**) aspect ratio and (**C**) roundness. Fold change was calculated for each biological replicate individually, resulting in four replicates for batch 1 and three replicates for batch 2. Data distribution was assessed using the Shapiro–Wilk test. Statistical comparisons were performed using unpaired two-tailed *t*-tests for normally distributed data and Mann–Whitney tests for non-normal distributions. Dots represent individual biological replicates (*n* = 3 per condition). Asterisks above bars indicate statistical significance compared to the control condition. Error bars represent standard deviation (SD). *p* < 0.05 (*), *p* < 0.01 (**), *p* < 0.001 (***), *p* < 0.0001 (****). Abbreviations: al = aligned; ran = random [30].

**Figure 6:**
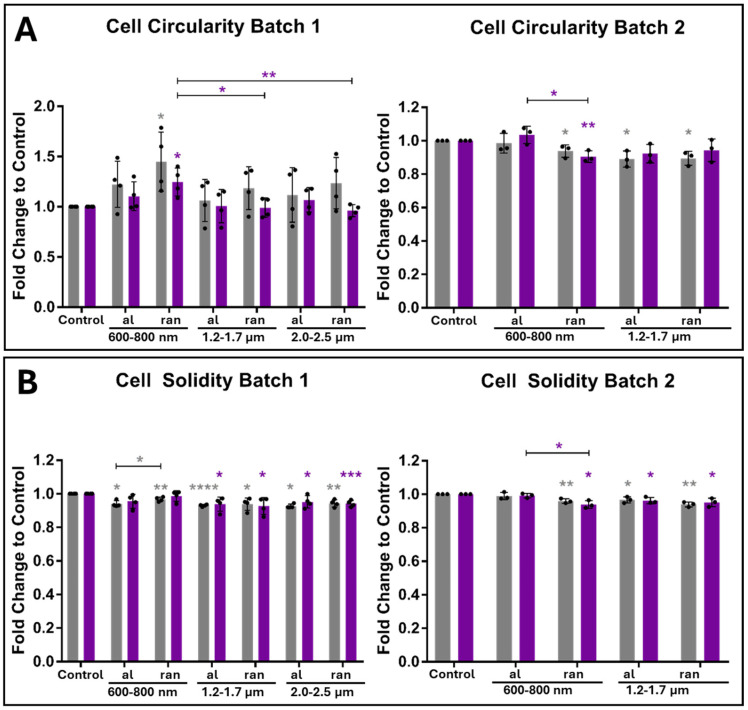
Temporal changes in ihGK cell morphology on various scaffold types compared to control cells at day 3 and day 7. Bar graphs illustrate the fold change in mean values of ihGK cell shape descriptors on different nanotopographic scaffolds, normalized to flat control surfaces, at day 3 and day 7. Quantified parameters include the following: (**A**) circularity and (**B**) solidity. Fold change was calculated for each biological replicate individually, resulting in four replicates for batch 1 and three replicates for batch 2. Data distribution was assessed using the Shapiro–Wilk test. Statistical comparisons were performed using unpaired two-tailed *t*-tests for normally distributed data and Mann–Whitney tests for non-normal distributions. Dots represent individual biological replicates (*n* = 3 per condition). Asterisks above bars indicate statistical significance compared to the control condition. Error bars represent standard deviation (SD). *p* < 0.05 (*), *p* < 0.01 (**), *p* < 0.001 (***), *p* < 0.0001 (****). Abbreviations: al = aligned; ran = random [30].

To facilitate comparison of these trends across batches despite inter-batch variability, we expressed each shape descriptor as a fold-change relative to the flat control for that batch (Figure 5 and Figure 6). This normalization confirmed that aligned scaffolds consistently induced more elongated cell morphologies (AR significantly above 1.0-fold of control; roundness and circularity below 1.0-fold of control), whereas random fiber scaffolds and control surfaces promoted a more rounded, spread-out cell shape with values near or slightly above the control baseline.

In summary, at day 7, scaffold-induced cell morphology trends were directionally consistent across both experimental batches, with aligned fiber scaffolds promoting elongated cell shapes and random or control surfaces yielding more rounded cells. Moreover, while batch-specific differences in absolute shape descriptor values were observed, the topography-driven trends remained consistent. In both batches, cells on aligned fiber scaffolds were more elongated (higher AR, lower circularity), whereas those on random fibers or planar controls remained more compact and rounded. Notably, aligned scaffolds with 1.2–1.7 µm fibers consistently supported the most elongated cell shapes across both batches. Thus, fiber orientation had a more pronounced effect on cell morphology than fiber diameter, although both parameters influenced cell shape. The full distribution of cell shape parameters for each condition is provided in Supplementary Figures S4–S7, illustrating that aligned fibers shift the cell population toward higher aspect ratios and lower circularity compared to random fibers. These consistent scaffold-specific effects underscore the robust topographical control of epithelial cell shape and highlight its relevance for biomaterial scaffold design. Cumulative distribution plots for each cell shape descriptor are provided in Supplementary Figures S4–S7 for both experimental batches (batch 1 and batch 2) at days 3 and 7, illustrating the full range of cell shape measurements under each condition.

### 3.3. Topography-Dependent Nuclear Shape Remodeling in Gingival Keratinocytes

Given the tight morphological coupling between cytoplasmic and nuclear shape in stratified epithelia, we next investigated whether scaffold topography affects nuclear geometry in ihGK cells. Nuclear morphology was quantified using the same shape descriptors as for the cell body, area, aspect ratio, roundness, circularity, and solidity via an ImageJ-based analysis pipeline (Table 3 and Table 4; Figure S8). Nuclear morphology is an established mechanosensitive readout: mechanical strain along aligned nanofibers can decrease nuclear area, whereas strain applied perpendicular to the fiber axis increases nuclear area [34]. Such deformations influence gene expression and cell fate [34]. The nuclear metrics (area, aspect ratio, roundness, circularity, solidity) reported here therefore provide insight into topography-dependent mechanotransduction.

In both scaffold batches, nuclei of ihGK cells exhibited a general trend toward reduced area and increased elongation after 7 days of culture (Table 4). This was particularly evident in batch 1, where aligned scaffolds consistently induced more elongated nuclear shapes (increased AR, decreased roundness), while random scaffolds maintained more rounded nuclei (Figure S9B). Interestingly, nuclear area was highest on random scaffolds at both time points, contrasting with control surfaces, where the smallest nuclear areas were observed (Figure S9A). The effect was attenuated but still partially recapitulated in batch 2, especially on 1.2–1.7 µm scaffolds.

Consistent with the trends measured by cell shape descriptors (Figure 3 and Figure 4), scaffold geometry markedly influenced nuclear shape. Across both 3-day and 7-day cultures, aligned fiber scaffolds induced a more elongated nuclear morphology than flat controls, evident as higher aspect ratio (AR) and lower roundness values (with the effect most pronounced in batch 1). For instance, by day 7 (Table 4), the average nuclear AR on medium-diameter aligned fibers reached ~1.6 in batch 1, versus ~1.3 on random fibers and planar controls, with a corresponding drop in nuclear roundness from ~0.78 on random/control surfaces to ~0.67 on aligned scaffolds. Nuclei on random-fiber scaffolds, in contrast, remained relatively round and even exhibited larger projected areas than those on aligned or control substrates (e.g., ~226 µm^2^ on random fibers vs. ~177 µm^2^ on control at 3 days; Table 3). While these topography-dependent shape differences were consistently observed in the first experimental batch, they were markedly attenuated in the second batch (Figure S9B,C), underscoring some inter-experimental variability. Despite pronounced shape remodeling, nuclear envelope integrity was largely maintained across conditions. Nuclear circularity and solidity values remained high (generally > 0.75 and > 0.92, respectively) on all surfaces, indicating that nuclei retained an intact, regular outline. Nevertheless, aligned fibers in batch 1 caused slight but significant reductions in both circularity and solidity relative to controls, suggesting a degree of nuclear reshaping, possibly linked to scaffold-induced cytoskeletal remodeling (Figure S9D,E).

Overall, these findings demonstrate that fiber orientation and diameter can modulate nuclear architecture in gingival keratinocytes in a time- and batch-dependent manner, paralleling the cell morphology changes. Crucially, the preservation of high nuclear boundary regularity implies that nuclear structural integrity was not compromised by these shape changes. Such scaffold-induced nuclear shape adaptations likely result from mechanical coupling between the cytoskeleton and nucleus and may carry functional consequences. Indeed, because alterations in nuclear morphology can reflect changes in chromatin organization, gene expression, or cell cycle status, we next investigated whether the observed nuclear shape differences were accompanied by changes in keratinocyte proliferation.

### 3.4. Aligned Topographies Sustain Proliferation While Random Fibers Suppress Expansion

**Figure 7:**
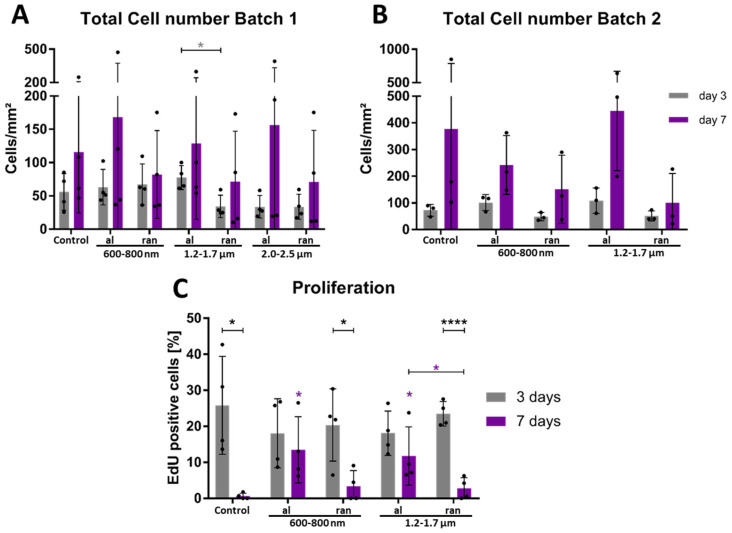
Quantification of ihGK cell density and proliferation activity on different scaffold surfaces across two batches and time points. (**A**) Cell density of ihGK cells cultured on various scaffold topographies in batch 1 (*n* = 4 biological replicates) was determined at day 3 and day 7. (**B**) Cell density of ihGK cells in batch 2 (*n* = 3 biological replicates) was quantified under the same conditions. For both (**A**,**B**), total cell counts per mm^2^ were calculated from fluorescence microscopy images using a Fiji/ImageJ-based nuclear segmentation workflow. Individual data points represent biological replicates and bar graphs illustrate the fold change in mean values. (**C**) Proliferation activity of ihGK cells cultured on different surface topographies was assessed at day 3 and day 7 using the Click-iT EdU assay. EdU-positive nuclei were quantified relative to the total number of nuclei using a Fiji/ImageJ-based analysis pipeline (*n* = 4 biological replicates). For all panels, data normality was evaluated using the Shapiro–Wilk test. Statistical comparisons were performed using unpaired two-tailed *t*-tests for normally distributed data and Mann–Whitney tests for non-normally distributed data. Asterisks above the bars indicate statistical significance relative to the control condition. Error bars represent standard deviation (SD). *p* < 0.05 (*), **** *p* < 0.0001. Abbreviations: al = aligned; ran = random [30].

To determine whether scaffold architecture affects epithelial cell proliferation, we quantified both total cell density and the fraction of actively proliferating ihGK cells after 3 and 7 days of culture. Cell numbers were derived from automated nuclear segmentation based on fluorescence images (Figure 7A,B), while proliferation rates were assessed via EdU incorporation (Figure 7C) and a Fiji/ImageJ-based detection pipeline (Figure S10).

Across both batches, ihGK cell numbers increased from day 3 to day 7 on all substrates, but the extent of accumulation varied with scaffold architecture (Figure 7). At day 3, cell densities were broadly similar across topographies; for example, in batch 1, the 1.2–1.7 µm aligned fibers actually had the highest cell density among the aligned conditions, underscoring the absence of a consistent pattern (Figure 7). By day 7, all aligned scaffolds supported higher cell densities than their random counterparts and the flat control, where the 1.2–1.7 µm aligned fibers were the lowest under all aligned conditions (Figure 7A), although the exact ranking of aligned conditions differed between batches (Figure 7A,B). These observations suggest that initial cell attachment and proliferation were comparable across topographies, whereas, over time, aligned fibers promoted sustained cell accumulation and random fibers were less conducive to cell growth.

To directly assess proliferation, EdU-based quantification was performed for batch 2 (Figure 7C). On day 3, control cells showed the highest fraction of EdU-positive nuclei (~26%), while aligned and random scaffolds exhibited moderately lower rates (18–23%). Interestingly, cells cultured on 600–800 nm random scaffolds displayed a transient proliferative boost at day 3 (~20%), indicating that disordered submicron topographies initially promote proliferation (Figure 7C). However, this effect was not sustained: by day 7, proliferation was nearly absent on control and random scaffolds (<4%), while aligned scaffolds retained substantial EdU activity (600–800 nm: ~13%, 1.2–1.7 µm: ~12%). The 1.2–1.7 µm aligned condition exhibited significantly higher proliferation than its random counterpart (Figure 7C). In addition, EdU-positive cells on aligned scaffolds frequently occurred in tightly clustered distributions, a growth behavior not observed on planar or random substrates. The decline in EdU-positive nuclei on random scaffolds by day 7 reflects a reduction in the fraction of cells entering S phase; the Click-iT EdU assay only labels cells actively synthesizing DNA. After an initial proliferative burst, cells on random scaffolds likely exit the cell cycle and commence differentiation [7], leading to decreased EdU labeling even as total cell density increases. This explanation reconciles the divergent trends observed for EdU incorporation and cell density.

Together, these findings indicate that scaffold alignment supports both higher cell densities and sustained proliferative activity over time, independently of fiber diameter. Random fiber architectures, by contrast, promote an early but transient proliferative response that transitions to a non-proliferative state by day 7. These results position fiber orientation as a critical design parameter for modulating epithelial cell expansion on bioengineered scaffolds. As scaffold geometry clearly modulated both cell shape and proliferation dynamics, we next examined its impact on keratinocyte differentiation.

### 3.5. Fiber Orientation and Diameter Drive Topography-Specific Differentiation Programs

To evaluate the impact of scaffold architecture on epithelial differentiation, basal, early, and terminal keratinocyte markers were quantified by droplet digital PCR (ddPCR) after 3 and 7 days of cultivation on batch 1 surfaces. Despite low RNA yields, particularly at early time points, transcript copy numbers were reliably quantified across all groups, with control and aligned scaffolds yielding the highest RNA concentrations.

**Figure 8:**
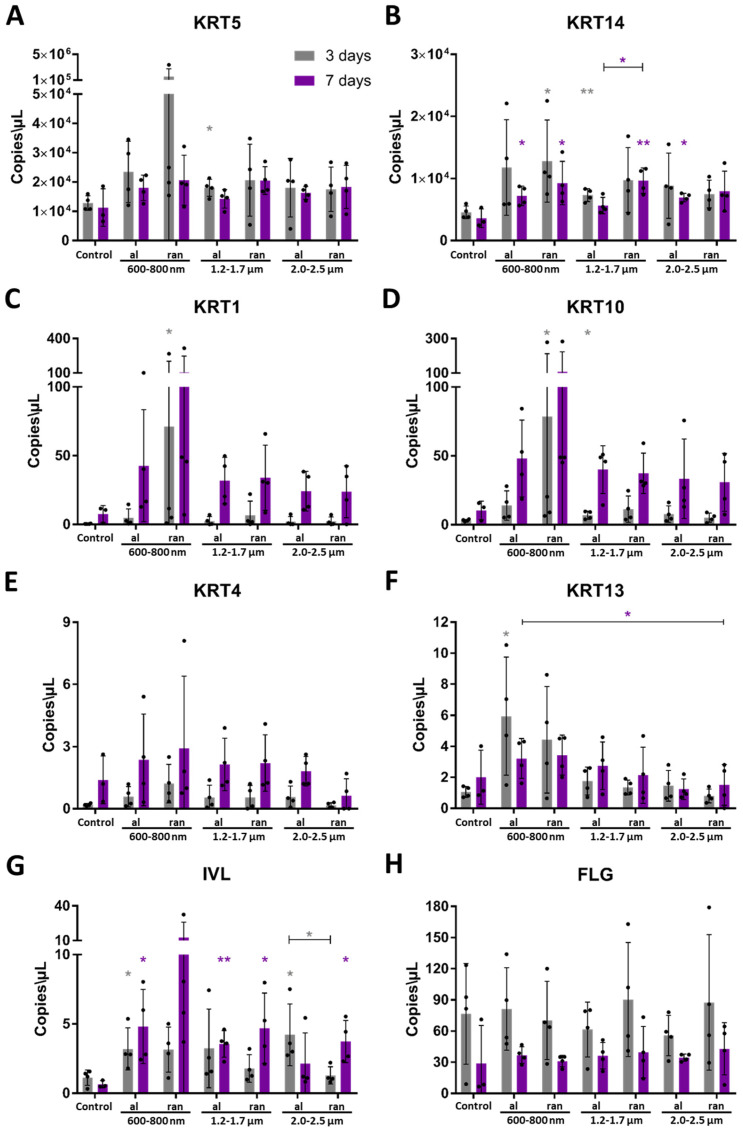
Expression analysis of basal, early and late differentiation markers in ihGK cells using droplet digital PCR (ddPCR) following cultivation on different scaffolds (batch 1). Basal cell marker expression (**A**) KRT5 and (**B**) KRT14, early differentiation marker expression (**C**) KRT1, (**D**) KRT10, (**E**) KRT4 and (**F**) KRT13, and late differentiation marker expression (**G**) IVL and (**H**) FLG in ihGK cells was quantified after 3 and 7 days of culture on batch 1 scaffolds using droplet digital PCR. Total RNA was isolated, reverse-transcribed into cDNA, and subjected to ddPCR. Gene copy numbers were measured in copies/µL. Each dot represents one biological replicate (*n* = 4) and bar graphs illustrate the fold change in mean values, except for the control condition at day 7 (*n* = 3). Due to limited RNA yield, only one technical replicate per sample was performed. Data normality was evaluated using the Shapiro–Wilk test. Statistical comparisons were made using unpaired two-tailed *t*-tests for normally distributed data or Mann–Whitney tests for non-normally distributed data. Asterisks above bars indicate significance compared to the control. Error bars represent standard deviation (SD). *p* < 0.05 (*), *p* < 0.01 (**). Abbreviations: al = aligned, ran = random [30].

Basal markers KRT5 and KRT14 showed markedly elevated expression in scaffold-cultured ihGK cells compared to controls (Figure 8A,B). The strongest expression was detected on 600–800 nm random scaffolds at day 3, with decreasing levels on larger fibers and a general decline over time, indicating exit from the basal phenotype.

At day 7, early differentiation markers KRT1, KRT10, KRT4, and KRT13 were upregulated on scaffold surfaces compared to control (Figure 8C–F). KRT1 and KRT10 showed the strongest upregulation, with maximal expression on 600–800 nm random scaffolds. In contrast, KRT4 and KRT13 exhibited overall lower transcript levels across all conditions, but still reached their highest expression on 600–800 nm random fibers, with expression decreasing as fiber diameter increased. Notably, KRT13 expression was similar on aligned and random fibers of the same diameter, indicating that fiber orientation has only a modest effect on this marker. These findings underscore that while small-diameter random fibers most strongly drive KRT1/10, KRT4 and KRT13 respond more moderately and are less sensitive to fiber orientation.

The terminal marker IVL was upregulated by day 7 on most surfaces, most prominently on 600–800 nm random scaffolds (Figure 8G). FLG expression exceeded IVL across all conditions and time points but showed minimal temporal variation (Figure 8H). Notably, random scaffolds promoted higher FLG expression at larger fiber diameters (1.2–2.5 µm), whereas aligned 600–800 nm scaffolds induced the highest FLG levels within that group. These opposing trends reflect the normal progression of keratinocytes from a proliferative basal state to a differentiated suprabasal phenotype. Basal keratins KRT5 and KRT14 are expressed in proliferative basal cells and are downregulated during differentiation, whereas suprabasal keratins (KRT1/KRT10 and KRT4/KRT13) and terminal markers (IVL, FLG) are induced as keratinocytes stratify [7]. Declining LMNB1 expression and changes in YAP1 activity also accompany differentiation and senescence [35,36]. Accordingly, the decrease in basal marker expression and increase in differentiation markers over time indicate progression from a proliferative to a differentiated state.

Together, these data demonstrate that scaffold architecture, particularly random alignment and reduced fiber diameter, modulates transcriptional programs associated with epithelial differentiation. Downregulation of basal markers alongside increased early and terminal gene expression over time confirms a topography-dependent progression of differentiation in ihGK cells. Protein-level analyses were subsequently performed to validate these findings.

### 3.6. Topographical Cues Shape Protein-Level Differentiation in Epithelial Cells

To assess whether transcriptomic trends are reflected at the protein level, expression intensities of key markers were quantified by semi-automated fluorescence image analysis after 3 and 7 days of culture on batch 2 scaffolds. Fluorescent intensities were extracted using a Fiji/ImageJ-based algorithm, allowing comparison of integrated protein levels normalized to control cells.

**Figure 9:**
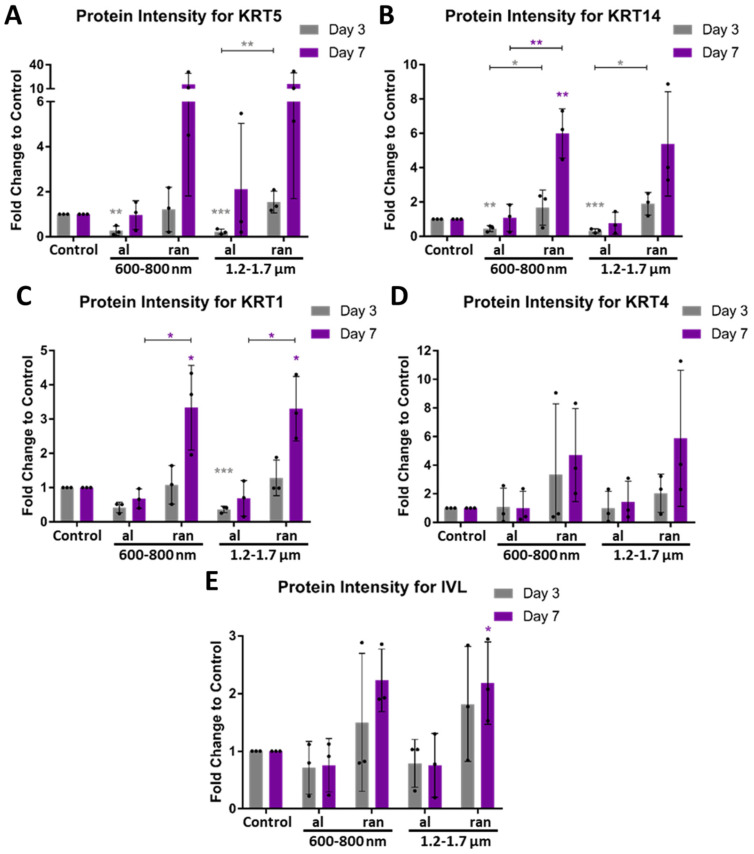
Quantitative analysis of basal and terminal differentiation marker protein expression in ihGK cells cultured on various scaffold surfaces after 3 and 7 days. (**A**) Fold change of KRT5, (**B**) KRT14, (**C**) KRT1 and (**D**) KRT4 protein expression in ihGK cells on different scaffold topographies, measured after 3 and 7 days, relative to control conditions. (**E**) Fold change of IVL protein expression, representing terminal differentiation, relative to control cells at each time point. Each dot represents an individual biological replicate (*n* = 3), and error bars indicate standard deviation (SD). Data were assessed for normality using the Shapiro–Wilk test. Statistical analysis was performed using unpaired two-tailed *t*-tests for normally distributed data or Mann–Whitney tests for non-normally distributed data. Asterisks above bars indicate significance relative to the control. *p* < 0.05 (*), *p* < 0.01 (**), *p* < 0.001 (***). Abbreviations: al = aligned; ran = random [30].

Basal markers KRT5 and KRT14 showed reduced intensities on aligned scaffolds, while ihGK cells on random scaffolds, especially with 600–800 nm fibers, exhibited higher expression levels at both time points (Figure 9A,B and Figure S11A). Significant upregulation was observed for KRT14 on 600–800 nm random scaffolds after 7 days. Notably, in contrast to ddPCR results (Figure 8A,B), protein levels tended to increase over time (Figure 9A,B).

Early differentiation markers, KRT1 and KRT4, followed a similar trend (Figure 9C,D and Figure S12A). Cells on aligned fibers displayed consistently lower protein levels compared to random scaffolds, with significantly higher intensities for KRT1 and by trend KRT4 intensities in ihGK cells on 600–800 nm and 1.2–1.7 µm random scaffolds after 7 days (Figure 9C,D).

Terminal differentiation marker IVL was strongly upregulated in ihGK cells on random scaffolds, particularly after 7 days (Figure 9E and Figure S13). Aligned scaffolds, by contrast, maintained protein levels similar to or below those of controls.

Altogether, these data confirm that random scaffold topographies, particularly with smaller fiber diameters, promote increased protein expression of basal, early, and terminal epithelial differentiation markers. This protein-level regulation aligns with transcriptional patterns observed under comparable conditions and reinforces fiber orientation and diameter as key determinants of epithelial cell differentiation state.

### 3.7. Batch-Resolved Analysis Reveals Consistent Scaffold-Guided Functional Outcomes

Scaffold architecture exerted reproducible effects on ihGK morphology, nuclear shape, gene/protein expression, and proliferation, as summarized in Figure S14 (batch 1) and Figure S15 (batch 2).

In batch 1, fiber orientation determined cell shape and nuclear morphology. Aligned scaffolds supported elongated cells and nuclei at both time points, while random scaffolds and control induced rounded shapes. Random 600–800 nm fibers promoted the largest nuclear area and highest RNA levels, including KRT5, KRT14, KRT1, KRT10, and IVL. Increasing fiber diameter reduced both cell area and gene expression across all orientations. A progressive decrease in cell and nuclear size from day 3 to day 7 was observed, accompanied by increased expression of early and terminal differentiation markers. Random scaffolds consistently induced higher RNA levels than aligned ones, with the strongest upregulation at 600–800 nm.

In batch 2, scaffold-induced responses were similar but less pronounced. Cells on aligned scaffolds showed reduced elongation compared to batch 1, consistent with denser fiber cross-linking. In contrast, random 1.2–1.7 µm scaffolds supported increased elongation due to straighter fiber segments. Cell and nuclear areas decreased over time. Protein expression was highest on random scaffolds, and lowest on aligned surfaces. At day 3, KRT5, KRT14, KRT1, and IVL were strongly expressed on random 1.2–1.7 µm fibers. By day 7, both random conditions supported persistent protein upregulation across all markers tested.

Proliferation was highest on control and random scaffolds at day 3, but declined to near zero by day 7. In contrast, aligned scaffolds retained >10% proliferation, suggesting that orientation modulates temporal proliferation dynamics.

Overall, aligned fibers supported proliferative maintenance, whereas random scaffolds promoted differentiation, gene as well as protein induction, and nuclear enlargement (Figures S14 and S15).

### 3.8. Basal Keratins (KRT5/14) Enable Topography-Dependent Epithelial Fate and Viability

The prominent up-regulation of KRT5 and KRT14 in ihGK cells cultured on scaffolds, particularly under random fiber conditions, coincided with enhanced expression of differentiation and adhesion markers. These observations prompted functional knock-down experiments to dissect the role of basal keratins in mediating scaffold-responsive cell fate.

Given the established interplay between basal keratins and mechanosensitive pathways such as FAK and YAP1 [37], our analysis focused on transcript-level changes as an initial readout of knockdown efficiency and scaffold-dependent regulatory responses. Protein-level analyses (e.g., subcellular YAP localization) were deliberately not included at this stage, as our aim was to assess whether KRT5/14 depletion broadly perturbs the expression of downstream effectors rather than to map their mechanistic function. This mRNA-centric approach allowed for a focused comparison across topographies and genes of interest, particularly in light of the substantial cell loss observed under knockdown conditions on random scaffolds (see below).

As a control, we profiled gene expression after 96 h on the scaffolds (without siRNA treatment) and confirmed the same trends: random fibers induced higher expression of basal KRT5/14 and differentiation markers KRT1, KRT4 and IVL (Figure S16), as well as adhesion genes ITGB1/ITGB3/FAK (Figure S17), compared to aligned fibers and flat surfaces. Mechanosensitive genes like YAP1 and LMNB1 did not significantly differ at the mRNA level across topographies (Figure S18).

qPCR analyses were performed at a single time point (96 h), which we selected as a compromise between knockdown efficacy and cell viability. Preliminary time-course experiments with ihGKs indicated that earlier RNA extraction (e.g., 48 h) yielded inconsistent transcript levels, while extended culture (>96 h) led to reduced cell integrity (own preliminary observation.

**Figure 10:**
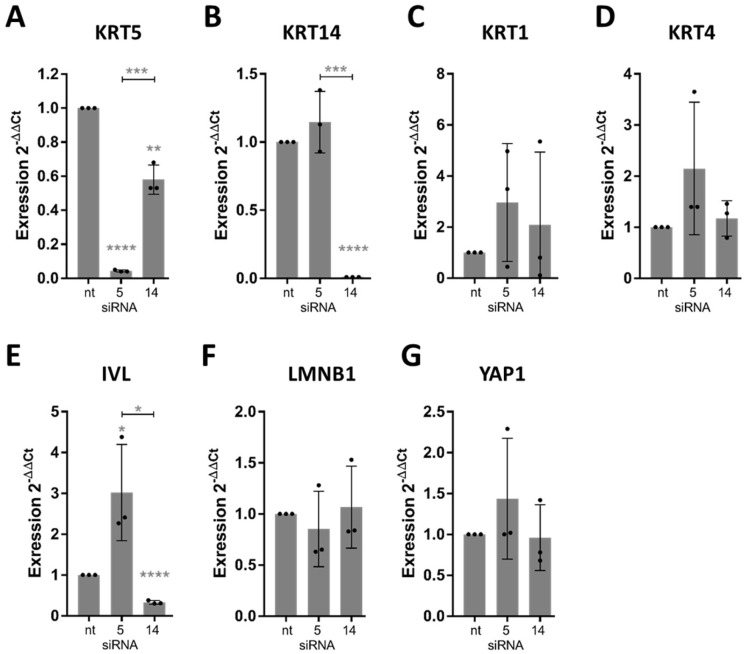
siRNA-mediated knockdown of KRT5 and KRT14 in ihGK cells cultured on control surfaces. ihGK cells were cultured for 24 h on flat control surfaces prior to treatment with siRNA targeting KRT5 and KRT14 (final concentration: 200 nM) for 72 h. Total RNA was isolated and subjected to quantitative PCR (qPCR) to assess expression levels of (**A**) KRT5, (**B**) KRT14, (**C**) KRT1, (**D**) KRT4, (**E**) IVL, (**F**) LMNB1, and (**G**) YAP1. Relative gene expression was normalized to non-targeting (nt) control-treated cells. Each dot represents one biological replicate (*n* = 3). Error bars indicate standard deviation (SD). Normality of the data was tested using the Shapiro–Wilk test. Statistical comparisons were made using unpaired two-tailed *t*-tests for normally distributed data and Mann–Whitney tests for non-normally distributed data. Asterisks above bars indicate statistical significance compared to the control group. *p* < 0.05 (*), *p* < 0.01 (**), *p* < 0.001 (***), *p* < 0.0001 (****) [30].

On control surfaces, KRT5 knock-down led to increased KRT1, KRT4, and IVL expression, while reducing LMNB1 and moderately increasing YAP1 (Figure 10). KRT14 knock-down reduced IVL and KRT5 expression while elevating KRT1 and KRT4. LMNB1 increased slightly, whereas YAP1 declined (Figure 10F,G).

**Figure 11:**
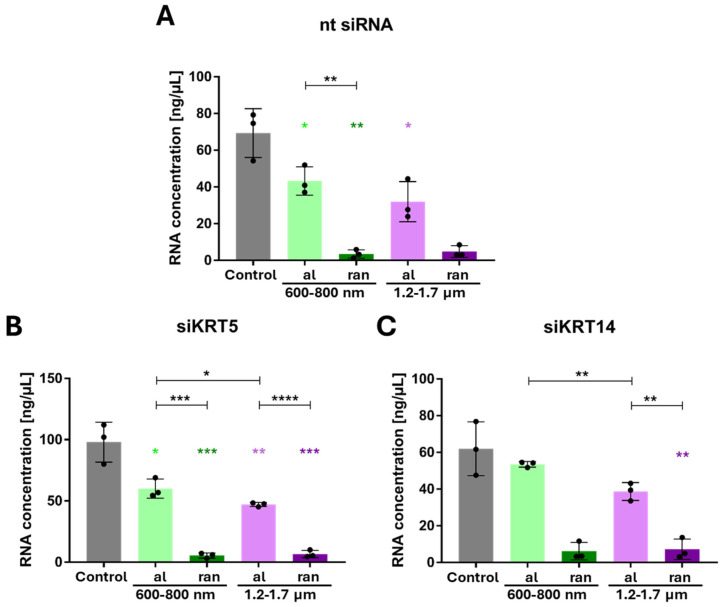
RNA concentrations of ihGK cells following siRNA treatment on different scaffold surfaces. ihGK cells were cultured for 24 h prior to treatment with (**A**) non-targeting siRNA (nt), (**B**) siRNA against KRT5 (siKRT5), or (**C**) siRNA against KRT14 (siKRT14) for 72 h. Total RNA was isolated, and concentrations were determined. Each data point represents a biological replicate (*n* = 3). Error bars indicate standard deviation (SD). Data normality was assessed using the Shapiro–Wilk test; statistical analysis was performed using unpaired two-tailed *t*-tests or Mann–Whitney tests, as appropriate. Connecting brackets show the specific groups compared. *p* < 0.05 (*), *p* < 0.01 (**), *p* < 0.001 (***), *p* < 0.0001 (****). Abbreviations: al = aligned, ran = random [30].

**Figure 12:**
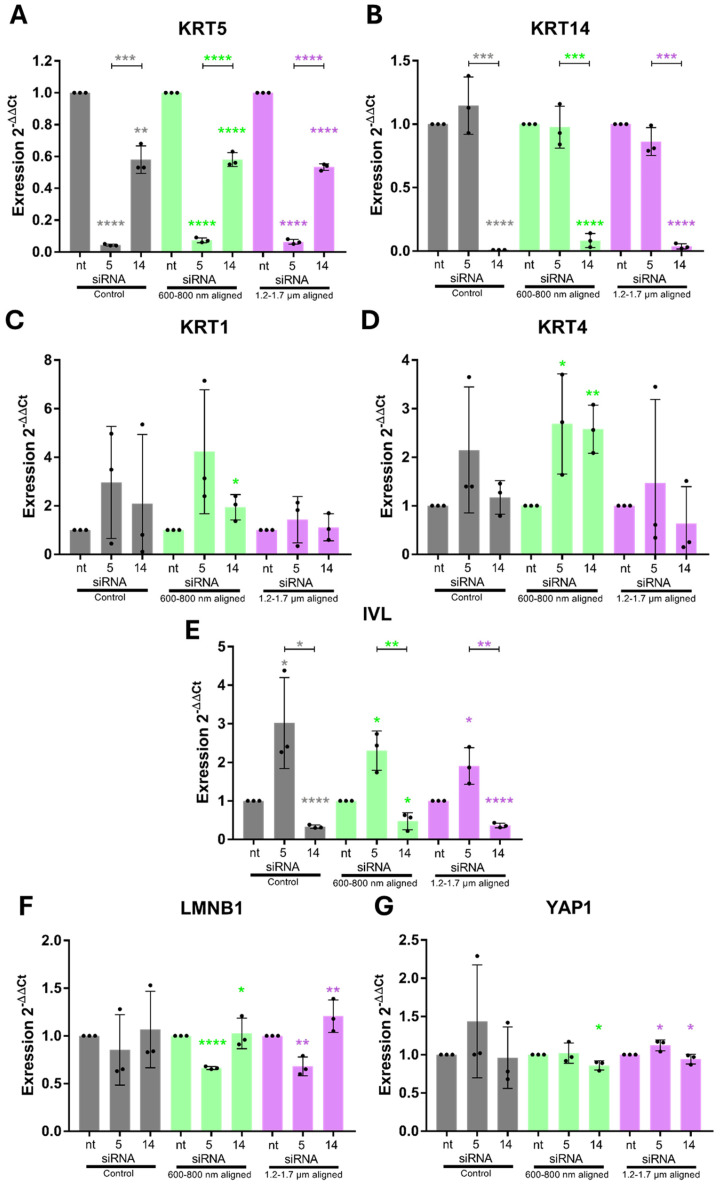
siRNA-mediated knockdown of KRT5 and KRT14 in ihGK cells on control and aligned scaffold surfaces. ihGK cells were cultured for 24 h prior to siRNA-mediated knockdown of KRT5 and KRT14, or treatment with non-targeting (nt) siRNA, followed by 72 h of incubation on either control or aligned surfaces. RNA was isolated and qPCR was performed to assess expression of (**A**) KRT5, (**B**) KRT14 (basal markers), (**C**) KRT1, (**D**) KRT4 (early differentiation markers), (**E**) IVL (terminal differentiation marker), (**F**) LMNB1 (nuclear integrity marker), (**G**) YAP1 (proliferation-associated co-transcriptional activator). Gene expression was normalized to nt siRNA-treated cells for each respective surface. Each dot represents a biological replicate (*n* = 3). Error bars show standard deviation (SD). Statistical analysis was based on the Shapiro–Wilk test for normality and either unpaired two-tailed *t*-test or Mann–Whitney test, as appropriate. Connecting brackets show the specific groups compared. *p* < 0.05 (*), *p* < 0.01 (**), *p* < 0.001 (***), *p* < 0.0001 (****). Abbreviations: al = aligned, ran = random [30].

On random scaffolds, siRNA-treated cells exhibited nearly complete loss of RNA content, indicating population collapse (Figure 11). Due to severe cell loss on random scaffolds following KRT5 and KRT14 knockdown, RNA yields from these conditions were below detection threshold and not suitable for downstream qPCR analysis. Consequently, differential gene expression was only assessed in aligned scaffold cultures, where both cell viability and RNA integrity were reliably maintained. In contrast, aligned scaffold conditions permitted RNA recovery and analysis. On aligned scaffolds, KRT5 knock-down induced significant up-regulation of KRT1 and KRT4 (particularly under 600–800 nm conditions), along with increased IVL expression (Figure 12C–E). LMNB1 was significantly reduced, while YAP1 expression increased, especially on 1.2–1.7 µm fibers (Figure 12F,G). KRT14 knock-down reduced IVL and KRT5 expression, increased LMNB1, and decreased YAP1 levels. These outcomes recapitulated findings from control conditions and highlighted differential diameter-dependent responses.

**Figure 13:**
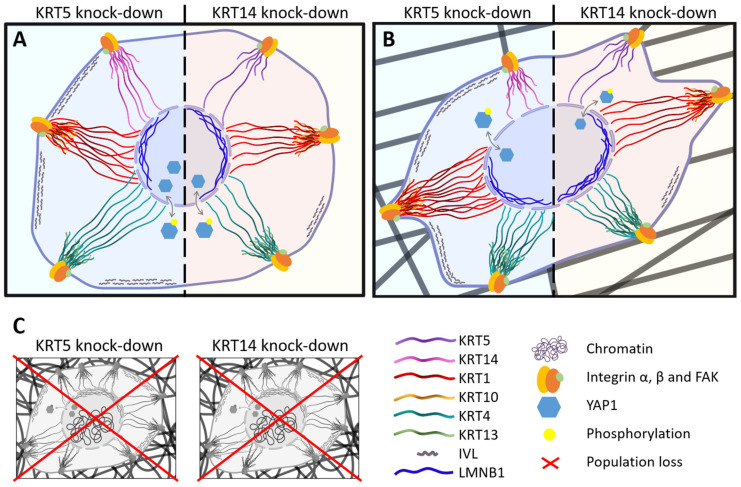
Schematic overview of ihGK cell responses to KRT5 and KRT14 knockdown on different scaffold surfaces. Illustrations depict the effects of siRNA-mediated knockdown of KRT5 and KRT14 on ihGK cells cultured on (**A**) flat control surfaces, (**B**) aligned scaffolds, and (**C**) random scaffolds. The visual representations are based on experimental results and show marker abundance for basal, early, and terminal differentiation (KRT5, KRT14, KRT1, KRT4, IVL), as well as nuclear integrity (LMNB1) and the transcriptional co-activator YAP1. YAP1 is depicted in both its nuclear (non-phosphorylated) and cytoplasmic (phosphorylated) forms, based on total YAP detection. Loss of the entire cell population is indicated for random scaffolds upon knockdown. Focal adhesion proteins are displayed at comparable levels across conditions, as they were not assessed in this context. Illustrations were created using BioRender.com and PowerPoint 2016 [30].

Collectively, the data establish that KRT5 maintains basal epithelial identity and protects premature differentiation, while KRT14 supports epithelial maturation and may modulate nuclear architecture in response to scaffold stimuli, as suggested by altered IVL and LMNB1 levels upon KRT14 knockdown. The inability of ihGK cells to persist on random scaffolds following keratin knock-down confirms that both proteins are indispensable for scaffold-induced epithelial homeostasis (Figure 13). In summary this implies that aligned fibers preserve a basal phenotype—cells stay alive and keep proliferating even if some keratins are lost—whereas random fibers drive differentiation and absolutely require KRT5/14 for cell survival

## 4. Discussion

The current study demonstrates that electrospun scaffold topography, defined by fiber orientation and diameter, exerts a profound influence on ihGK morphology, nuclear architecture, proliferative activity, and differentiation. Through a multimodal approach integrating qualitative imaging, morphometric analysis, transcriptomic and proteomic profiling, and functional RNAi experiments, we provide a comprehensive mechanobiological characterization of how scaffold features regulate epithelial cell fate. These findings guide the rational design of next-generation functional biomaterials for mucosal regeneration.

Aligned fiber scaffolds, particularly within the 1.2–1.7 µm diameter range, promoted elongated cell and nuclear morphologies. These topographies induced increased aspect ratio and reduced roundness, morphological features commonly associated with cytoskeletal polarization and focal adhesion formation in response to topographic guidance [17,38,39]. The persistence of these features across time points and scaffold batches underscores the robustness of topography-driven cell shape modulation. These findings are aligned with prior studies showing that fiber-guided alignment modulates cytoskeletal tension and nuclear form via LINC complex-coupled [40,41].

In contrast, random scaffolds induced spherical cell shapes and larger nuclei. This geometry was associated with an initial increase in proliferation at day 3, followed by a marked decline by day 7. This biphasic pattern coincided with the upregulation of differentiation markers such as IVL and KRT1/10, suggesting a transient proliferative burst that precedes fate commitment. This behavior may reflect an early adaptive response to disordered topographies, potentially mediated by actin remodeling and stress fiber disorganization [42,43]. Despite nuclear reshaping, structural integrity remained preserved, as indicated by high circularity and solidity scores. The subtle remodeling of nuclear contours on aligned scaffolds suggests actin-mediated mechanotransduction at the nuclear envelope [44,45].

Functionally, aligned scaffolds sustained proliferation up to day 7, in contrast to random and planar surfaces. EdU-positive cells on aligned scaffolds frequently appeared in closely associated arrangements, indicating localized growth patterns not observed on planar or random substrates. Although stratification was not formally quantified, this spatial proximity may represent an early stage of epithelial organization. While fully stratified layering was not detected, these localized clusters could reflect initial steps toward architectural self-assembly. These findings support the hypothesis that aligned fiber orientation fosters a microenvironment conducive to the preservation of basal keratinocyte identity and sustained proliferation [18,46]. Taken together, the observed morphometric signatures reflect scaffold-specific biophysical constraints that correlate with divergent epithelial outcomes. Elongated cell shapes on aligned scaffolds align with sustained proliferation and basal marker expression, whereas compact morphologies on random scaffolds parallel transcriptional profiles indicative of differentiation. These structure-function associations underscore the relevance of quantitative shape descriptors as readouts of topography-driven epithelial state transitions.

Gene and protein expression analyses confirmed scaffold-driven modulation of epithelial differentiation states. Random 600–800 nm fibers induced robust expression of basal (KRT5, KRT14), early (KRT1, KRT4), and terminal (IVL) markers. In contrast, aligned scaffolds suppressed this transcriptional program, supporting a basal-like phenotype. These fate transitions are consistent with prior findings that nanofiber alignment preserves undifferentiated states, while disordered random topographies are favorable in maturation-related epidermal epithelization during skin wound healing [47,48]. The simultaneous upregulation of basal and suprabasal markers on random scaffolds may indicate a transient intermediate state in which mechanical stress initiates differentiation priming while basal identity is still retained.

Taken together, the presented data establish a direct link between scaffold topography, defined by fiber diameter and alignment, and the regulation of epithelial cell morphology, proliferation, and differentiation. Aligned nanofiber scaffolds, particularly in the 600–800 nm and 1.2–1.7 µm range, reproducibly induced elongated cell and nuclear shapes, which were paralleled by elevated proliferation rates and increased total cell numbers over time. In contrast, random scaffolds, especially those with smaller diameters, promoted transcriptional activation of early and terminal differentiation markers, despite lower cell densities. The tight spatial coupling between cell elongation and nuclear geometry observed on aligned scaffolds indicates that topographical cues are effectively transmitted to the nucleus, possibly through cytoskeletal mechanotransduction, thereby influencing chromatin organization and gene expression profiles. This hypothesis is supported by the temporal progression from high basal keratin expression (KRT5, KRT14) toward suprabasal and terminal differentiation markers (KRT1, KRT10, IVL, FLG) across scaffold conditions, with random topographies accelerating differentiation-associated gene expression.

Our results also demonstrate that ihGK cells respond to scaffold geometry in a manner reminiscent of primary oral keratinocytes. The expression profile of native gingival epithelia, including key basal and suprabasal differentiation markers, was faithfully recapitulated, and previous studies have shown that these cells are capable of forming stratified epithelial layers in vitro under organotypic conditions [22]. Thus, the use of ihGK should not be viewed as a limitation but rather as a validated and reproducible system for dissecting topography-responsive epithelial fate.

The essential role of basal keratins in scaffold-induced cell fate became evident upon knockdown of KRT5 and KRT14. Silencing KRT5 efficiently depleted its transcripts and yielded a robust pro-differentiation response: suprabasal markers KRT1 and KRT4 were markedly upregulated, particularly on the 600–800 nm aligned fibers, and the terminal marker IVL rose substantially, while LMNB1 levels fell and YAP1 increased, suggesting weakened nuclear integrity and heightened mechanotransduction.

In contrast, KRT14 knockdown had subtler, context-dependent effects: it significantly reduced KRT5 expression, induced significant increase in KRT1 and KRT4 on 600–800 nm fibers, here restricted to the thinnest fibers, since on thicker fibers, 1.2–1.7 µm KRT4 is down-regulated, and IVL expression was reduced significantly on both aligned configurations as well. These differences indicate that KRT5 is the dominant regulator of the basal state and differentiation suppression, whereas KRT14 plays a more supportive role [7,49,50]. Notably, both KRT5 and KRT14 knockdowns caused a dramatic reduction in RNA yield on random (disordered) scaffolds, whereas aligned scaffolds of both diameters maintained moderate RNA concentrations. This indicates that the vulnerability arises from fiber orientation rather than fiber size. Although cell viability was not directly assessed, the near-zero RNA levels on random scaffolds are consistent with extensive cell loss [51,52]. By contrast, the aligned 600–800 nm scaffolds exhibited even higher RNA concentrations than the 1.2–1.7 µm aligned scaffolds, confirming that smaller diameters per se do not compromise RNA integrity.

In more detail, we identified topography-dependent modulation of nuclear regulators LMNB1 and YAP1. LMNB1 expression was modestly elevated under differentiation-promoting conditions and decreased upon KRT5 depletion, particularly on aligned scaffolds. This pattern suggests that LMNB1 responds to cytoskeletal tension and may serve as a readout of nuclear envelope strain. In contrast, KRT14 knockdown modestly increased LMNB1 expression, possibly reflecting compensatory stiffening in response to filament destabilization. YAP1 transcript levels remained largely unchanged across topographies and knockdowns, supporting the hypothesis that mechanotransduction at this level is regulated post-transcriptionally, particularly via nuclear translocation in response to mechanical stimuli [11]. While we did not explicitly investigate subcellular YAP localization, our data demonstrate a stable YAP1 transcript baseline across all scaffold conditions. This indicates that any scaffold-induced functional differences in YAP signaling are unlikely to be attributable to differential YAP1 gene expression.

Although the overall trends were consistent between batches, minor batch-to-batch variability was noted in certain outcomes (e.g., nuclear morphology metrics and RNA yields). These differences likely stem from inherent inconsistencies in the fabrication process, as the scaffolds were manufactured by an external. Indeed, SEM imaging (Figure S19) and fiber diameter measurements (Table S1) for the two batches confirmed clear differences in fiber configuration and diameter distribution. To address this variability, we validated key scaffold-induced biological effects across both batches. Observing consistent cell responses in two independent batches strengthens confidence in the robustness and generalizability of the findings.

Based on the complementary effects observed, we propose a layered “scaffold sandwich” architecture integrating proliferative and differentiating topographies. An aligned fiber layer (1.2–1.7 µm) would support the maintenance of basal keratinocyte identity during early wound closure, while an apical random nanofiber layer (600–800 nm) would subsequently induce differentiation-related stratification and barrier formation. This spatial orchestration mirrors native epithelial zonation and offers a design principle for future functional biomaterials [53]. Similar multilayered systems have already been explored in dermal and mucosal regeneration [54].

These findings underscore that even subtle changes in cell and nuclear shape can have biologically meaningful consequences in epithelial cells. Cell morphology adapts to mechanical cues and influences key behaviors such as proliferation, migration and differentiation. Small increases in cell spread area, for instance, have been linked to cell-cycle entry: in epithelial monolayers, expansion of cell area beyond a threshold triggers S-phase entry, whereas smaller cells remain in G1 [33]. Slight nuclear deformations can alter mechanotransduction and gene-expression programs, because perinuclear forces that deform the nucleus widen nuclear pores and enhance YAP/TAZ nuclear import [55]. In our study, the modest but consistent elongation of cells and nuclei on aligned fibers was accompanied by sustained proliferation and high basal keratin expression, whereas comparatively rounder cell shapes on random fibers coincided with up-regulation of differentiation markers. Fiber alignment proved to be a more influential determinant of morphology than fiber diameter: across batches and time points, aligned scaffolds induced higher aspect ratios and lower roundness than random scaffolds, independent of fiber size. These topography-induced morphological signatures correlated with divergent epithelial outcomes—elongated cells/nuclei on aligned scaffolds exhibited a basal, progenitor-like phenotype, whereas more spread, flattened cells on random scaffolds exhibited a differentiation-prone phenotype. This alignment between shape and function supports the notion that quantitative shape descriptors are informative readouts of cell state. Indeed, the cumulative distribution analyses in Supplementary Figures S4–S9 confirm that scaffold architecture consistently biases cell and nuclear morphology, providing a mechanistic link between fiber topography and keratinocyte fate.

Since no mechanical (tensile) testing was performed on the PCL fiber mats in the current study, we acknowledge that scaffold mechanical properties (e.g., stiffness, elasticity) can influence cellular responses and should be considered in future analyses [14,56]. Indeed, variations in fiber alignment or diameter may also affect the bulk mechanical stiffness of the mats, which in turn could contribute to the observed differences in cell behavior. Assessing the scaffold Young’s modulus or tensile strength was beyond the scope of this work; however, mechanical characterization of the fiber mats is recommended as future work to correlate scaffold stiffness with biological outcomes [14]. This perspective is supported by recent literature calling for thorough mechanical evaluation of electrospun scaffolds to optimize design parameters [14].

Rather than calling for specific methodological expansions, we emphasize here that our data provide a robust platform for future studies exploring biomarker dynamics under regenerative conditions. In this context, scaffold-induced modulation of keratin expression and nuclear mechanics offers a solid foundation for identifying molecular signatures relevant to epithelial homeostasis and restoration. Our approach delineates the biomechanical needs of keratinocytes during regeneration, independent of added cell types or complex co-culture systems.

## 5. Conclusions

This study establishes that fiber architecture, orientation and diameter plays a pivotal role in directing gingival keratinocyte fate. Aligned fiber scaffolds, particularly those with medium fiber diameters (~1.2–1.7 µm), induced the most pronounced cell and nuclear elongation, whereas random fiber scaffolds (especially with 600–800 nm fibers) produced more spread-out, rounded cell morphologies. Aligned topographies also sustained keratinocyte proliferation over time, in contrast to random fiber mats that elicited an initial proliferative burst at day 3, followed by a decline by day 7. In terms of differentiation, random fiber cultures, notably on the smallest fiber diameter, upregulated basal keratins (KRT5, KRT14) and early differentiation markers (KRT1, KRT10, KRT4, KRT13) relative to aligned fibers; accordingly, protein levels of involucrin (IVL) and filaggrin (FLG) were higher in cells on random mats than on aligned mats. Finally, KRT5/14 knockdown experiments revealed a striking scaffold-dependent viability difference. On random fiber scaffolds, loss of KRT5/14 drastically reduced cell viability, whereas on aligned fiber scaffolds cells remained viable despite KRT5/14 suppression. These insights define scaffold architecture as a programmable determinant of epithelial fate, advancing the development of next-generation functional biomaterials for oral tissue regeneration. The proposed “scaffold sandwich” configuration offers a modular framework to sequentially guide proliferation and differentiation, paving the way for tailored regenerative therapies in mucosal tissue restoration.

## Figures and Tables

**Table 1:** Comparison of mean ± standard deviation (SD) values for shape descriptors of ihGK cells after 3 days of cultivation on the tested surfaces in batch 1 (*n* = 4) and batch 2 (*n* = 3), X = no data. Full distribution plots of each shape descriptor at day 3 are provided in Supplementary Figure S4 (batch 1) and Figure S5 (batch 2).

3D		600–800 nm		1.2–1.7 µm		2.0–2.5 µm		Control
		Aligned	Random	Aligned	Random	Aligned	Random	
**Area [µm^2^]**	Batch 1	551.51 ± 300.79	509.81 ± 342.28	674.32 ± 382.40	729.64 ± 451.64	588.84 ± 390.81	689.53 ± 412.46	921.20 ± 587.75
	Batch 2	456.96 ± 255.96	518.85 ± 326.46	493.14 ± 270.28	566.66 ± 360.42	X	X	716.39 ± 457.79
**Aspect Ratio**	Batch 1	2.62 ± 1.32	1.80 ± 0.69	3.17 ± 1.71	1.72 ± 0.63	3.04 ± 1.79	1.76 ± 0.65	1.78 ± 0.84
	Batch 2	1.98 ± 0.83	1.85 ± 0.71	2.21 ± 0.99	1.93 ± 0.18	X	X	1.88 ± 0.74
**Roundness**	Batch 1	0.47 ± 0.19	0.61 ± 0.17	0.40 ± 0.19	0.64 ± 0.17	0.43 ± 0.20	0.63 ± 0.17	0.64 ± 0.19
	Batch 2	0.57 ± 0.18	0.60 ± 0.17	0.52 ± 0.18	0.59 ± 0.18	X	X	0.59 ± 0.17
**Circularity**	Batch 1	0.34 ± 0.13	0.41 ± 0.14	0.30 ± 0.12	0.33 ± 0.13	0.33 ± 0.15	0.34 ± 0.13	0.28 ± 0.11
	Batch 2	0.52 ± 0.14	0.50 ± 0.15	0.47 ± 0.14	0.47 ± 0.16	X	X	0.53 ± 0.12
**Solidity**	Batch 1	0.80 ± 0.10	0.82 ± 0.10	0.79 ± 0.10	0.79 ± 0.10	0.79 ± 0.12	0.80 ± 0.10	0.85 ± 0.07
	Batch 2	0.82 ± 0.07	0.80 ± 0.09	0.80 ± 0.08	0.78 ± 0.10	X	X	0.83 ± 0.07

**Table 2:** Comparison of mean ± standard deviation (SD) values for shape descriptors of ihGK cells after 7 days of cultivation on the tested surfaces in batch 1 (*n* = 4) and batch 2 (*n* = 3), X = no data. Full distribution plots of each shape descriptor at day 7 are provided in Supplementary Figure S6 (batch 1) and Figure S7 (batch 2).

7D		600–800 nm		1.2–1.7 µm		2.0–2.5 µm		Control
		Aligned	Random	Aligned	Random	Aligned	Random	
**Area [µm^2^]**	Batch 1	534.98 ± 540.04	510.61 ± 628.00	627.15 ± 560.99	694.12 ± 560.54	422.46 ± 406.45	612.19 ± 518.14	620.50 ± 593.24
	Batch2	361.80 ± 213.97	484.82 ± 401.39	421.67 ± 228.99	424.64 ± 404.54	X	X	630.37 ± 513.42
**AR**	Batch 1	2.75 ±1.52	1.81 ± 0.79	3.30 ±3.53	1.86 ± 0.81	2.75 ± 1.47	1.94 ± 0.87	1.79 ± 0.68
	Batch2	1.87 ± 0.74	1.78 ± 0.62	2.14 ± 0.98	1.97 ± 0.81	X	X	1.75 ± 0.64
**Roundness**	Batch 1	0.46 ± 0.21	0.62 ± 0.18	0.46 ± 0.21	0.61 ± 0.19	0.46 ± 0.20	0.59 ± 0.19	0.62 ± 0.17
	Batch2	0.60 ± 0.18	0.61 ± 0.17	0.54 ± 0.18	0.57 ± 0.18	X	X	0.62 ± 0.17
**Circularity**	Batch 1	0.35 ± 0.16	0.41 ± 0.16	0.31 ± 0.15	0.34 ± 0.15	0.33 ± 0.14	0.32 ± 0.12	0.32 ± 0.12
	Batch2	0.54 ± 0.13	0.47 ± 0.14	0.48 ± 0.14	0.48 ± 0.15	X	X	0.54 ± 0.12
**Solidity**	Batch 1	0.81 ± 0.10	0.83 ± 0.10	0.79 ± 0.12	0.79 ± 0.10	0.79 ± 0.12	0.77 ± 0.11	0.82 ± 0.08
	Batch2	0.82 ± 0.07	0.77 ± 0.09	0.80 ± 0.08	0.78 ± 0.10	X	X	0.83 ± 0.07

**Table 3:** Nucleus shape descriptor values (mean ± SD) of ihGK cells cultivated for 3 days on various surfaces, comparing batch 1 (*n* = 4) and batch 2 (*n* = 3), X = no data.

3D		600–800 nm		1.2–1.7 µm		2.0–2.5 µm		Control
		Aligned	Random	Aligned	Random	Aligned	Random	
**Area [µm^2^]**	Batch 1	195.55 ± 83.63	214.59 ± 98.93	175.82 ± 85.92	226.41 ± 106.81	191.06 ± 88.59	229.16 ± 101.25	177.36 ± 78.38
	Batch2	214.17 ± 96.40	254.85 ± 108.65	214.97 ± 92.22	228.40 ± 103.24	X	X	181.18 ± 84.00
**AR**	Batch 1	1.47 ± 0.37	1.33 ± 0.25	1.55 ± 0.39	1.33 ± 0.24	1.52 ± 0.46	1.35 ± 0.24	1.25 ± 0.20
	Batch2	1.38 ± 0.30	1.39 ± 0.27	1.44 ± 0.42	1.44 ± 0.37	X	X	1.36 ± 0.32
**Roundness**	Batch 1	0.71 ± 0.13	0.77 ± 0.12	0.68 ± 0.14	0.77 ± 0.12	0.70 ± 0.15	0.76 ± 0.12	0.82 ± 0.11
	Batch2	0.75 ± 0.13	0.74 ± 0.12	0.72 ± 0.13	0.73 ± 0.13	X	X	0.76 ± 0.13
**Circularity**	Batch 1	0.79 ± 0.08	0.79 ± 0.08	0.74 ± 0.12	0.77 ± 0.09	0.73 ± 0.10	0.76 ± 0.09	0.83 ± 0.07
	Batch2	0.81 ± 0.08	0.77 ± 0.09	0.81 ± 0.08	0.77 ± 0.09	X	X	0.80 ± 0.11
**Solidity**	Batch 1	0.96 ± 0.02	0.95 ± 0.02	0.94 ± 0.4	0.95 ± 0.02	0.95 ± 0.03	0.95 ± 0.02	0.96 ± 0.02
	Batch2	0.93 ± 0.03	0.92 ± 0.03	0.93 ± 0.02	0.92 ± 0.03	X	X	0.93 ± 0.03

**Table 4:** Nucleus shape descriptor values (mean ± SD) of ihGK cells cultivated for 7 days on various surfaces, comparing batch 1 (*n* = 4) and batch 2 (*n* = 3), X = no data.

7D		600–800 nm		1.2–1.7 µm		2.0–2.5 µm		Control
		Aligned	Random	Aligned	Random	Aligned	Random	
**Area [µm^2^]**	Batch 1	142.60 ± 76.24	175.97 ± 80.51	166.82 ± 92.11	169.38 ± 82.43	145.51 ± 76.74	168.42 ± 82.35	147.81 ± 64.90
	Batch2	178.34 ± 89.83	205.13 ± 82.93	157.58 ± 74.25	189.88 ± 89.88	X	X	144.44 ± 60.35
**AR**	Batch 1	1.55± 0.39	1.36 ± 0.26	1.57 ± 0.41	1.32 ± 0.23	1.65 ± 0.50	1.37 ± 0.26	1.32 ± 0.27
	Batch2	1.37 ± 0.27	1.34 ± 0.24	1.46 ± 0.34	1.46 ± 0.30	X	X	1.37 ± 0.31
**Roundness**	Batch 1	0.68 ± 0.14	0.76 ± 0.12	0.67 ± 0.15	0.78 ± 0.11	0.65 ± 0.16	0.75 ± 0.12	0.78 ± 0.12
	Batch2	0.75 ± 0.12	0.77 ± 0.12	0.72 ± 0.14	0.71 ± 0.13	X	X	0.76 ± 0.13
**Circularity**	Batch 1	0.77 ± 0.09	0.79 ± 0.09	0.74 ± 0.09	0.80 ± 0.09	0.71 ± 0.11	0.78 ± 0.10	0.83 ± 0.08
	Batch2	0.83 ± 0.08	0.83 ± 0.07	0.83 ± 0.08	0.79 ± 0.08	X	X	0.81 ± 0.10
**Solidity**	Batch 1	0.95 ± 0.02	0.95 ± 0.02	0.94 ± 0.3	0.95 ± 0.02	0.94 ± 0.03	0.95 ± 0.02	0.96 ± 0.01
	Batch2	0.93 ± 0.02	0.93 ± 0.02	0.93 ± 0.02	0.92 ± 0.02	X	X	0.92 ± 0.02

## Data Availability

The original contributions presented in this study are included in the article/Supplementary Materials. Further inquiries can be directed to the corresponding author.

## Supplementary Materials

The following supporting information can be downloaded at https://www.mdpi.com/article/10.3390/cells15030300/s1. Figure S1: ImageJ/Fiji-based workflow for quantitative analysis of cell morphology in ihGK cells cultured on aligned scaffolds. Figure S2: Semi-automated Fiji/ImageJ-based workflow for morphological analysis of ihGK cells cultured on 600–800 nm aligned scaffolds (batch 2) for three days. Figure S3: Quantitative shape descriptors used for morphometric analysis of ihGK cells. Figure S4: Cumulative distribution plots of ihGK cell shape descriptors after three days of culture on different scaffold topographies (batch 1). Figure S5: Cumulative distribution analysis of ihGK cell shape descriptors after three days of culture on different scaffold types (batch 2). Figure S6: Cumulative distribution plots of ihGK cell shape descriptors after seven days of culture on different scaffold topographies (batch 1). Figure S7: Cumulative distribution analysis of ihGK cell shape descriptors after seven days of culture on different scaffold architectures (batch 2). Figure S8: Mean values of nuclear shape descriptors of ihGK cells cultured on different scaffold topographies at day 3 and day 7 for batch 1 and batch 2. Figure S9: Temporal changes in nuclear shape descriptors of ihGK cells on different scaffold architectures, normalized to control conditions at day 3 and day 7. Figure S10: Fiji/ImageJ-based image analysis workflow for quantifying proliferation activity in ihGK cells using EdU incorporation. Figure S11: Basal cell marker expression in ihGK cells cultured on different scaffold surfaces after 3 and 7 days. Figure S12: Expression of early differentiation markers KRT1 and KRT4 in ihGK cells cultured on different scaffold surfaces after 3 and 7 days. Figure S13: Expression of the terminal differentiation marker IVL in ihGK cells cultured on different scaffold surfaces after 3 and 7 days. Figure S14: Schematic illustration of ihGK cell responses to different scaffold topographies after 3 and 7 days of culture (batch 1). Figure S15: Schematic overview of ihGK cell behavior on various scaffold surfaces after 3 and 7 days of culture (batch 2). Figure S16: Expression of differentiation markers in ihGK cells cultured for 96 h without treatment on various scaffold surfaces (batch 2). Figure S17: Expression of adhesion-related markers in ihGK cells cultured for 96 h without treatment on various scaffold surfaces (batch 2). Figure S18: Gene expression of LMNB1 and YAP1 in ihGK cells after 96 h of culture on different scaffold surfaces (batch 2). Figure S19: Scanning electron microscopy (SEM) analysis of scaffold and control surfaces without ihGK cells. Table S1: Scaffold diameter means ± SD of batch 1 and 2 scaffolds.

## Abbreviations

The following abbreviations are used in this manuscript:

AR	Aspect ratio (length/width measurement of cell shape)
C	Circularity (cell shape descriptor)
ddPCR	Droplet digital polymerase chain reaction
DMEM	Dulbecco’s Modified Eagle Medium
EdU	5-Ethynyl-2′-deoxyuridine (proliferation marker)
FAK	Focal adhesion kinase
FLG	Filaggrin (terminal differentiation marker)
ihGK	Immortalized human gingival keratinocytes
IVL	Involucrin (differentiation marker)
ITGB1	Integrin beta 1
ITGB3	Integrin beta 3
KRT1	Keratin 1 (early differentiation marker)
KRT4	Keratin 4 (early differentiation marker)
KRT5	Keratin 5 (basal keratin)
KRT10	Keratin 10 (early differentiation marker)
KRT13	Keratin 13 (early differentiation marker)
KRT14	Keratin 14 (basal keratin)
LMNB1	Lamin B1 (nuclear lamina protein)
PCL	Poly(ε-caprolactone) (electrospun scaffold material)
R	Roundness (cell shape descriptor)
S	Solidity (cell shape descriptor)
SEM	Scanning electron microscopy
siRNA	Small interfering RNA
YAP1	Yes-associated protein 1 (mechanosensitive transcriptional effector)

## References

[1] Mancini L., Romandini M., Fratini A., Americo L.M., Panda S., Marchetti E. (2021). Biomaterials for periodontal and peri-implant regeneration. Materials.

[2] Sevari S.P., Ansari S., Moshaverinia A. (2021). A narrative overview of utilizing biomaterials to recapitulate the salient regenerative features of dental-derived mesenchymal stem cells. Int. J. Oral Sci..

[3] Bouameur J.-E., Favre B., Fontao L., Lingasamy P., Begré N., Borradori L. (2014). Interaction of plectin with keratins 5 and 14: Dependence on several plectin domains and keratin quaternary structure. J. Investig. Dermatol..

[4] Ewald C.Y., Nyström A. (2023). Mechanotransduction through hemidesmosomes during aging and longevity. J. Cell Sci..

[5] Seltmann K., Fritsch A.W., Käs J.A., Magin T.M. (2013). Keratins significantly contribute to cell stiffness and impact invasive behavior. Proc. Natl. Acad. Sci. USA.

[6] Steinert P.M. (1996). Intermediate filaments in health and disease. Exp. Mol. Med..

[7] Alam H., Sehgal L., Kundu S.T., Dalal S.N., Vaidya M.M. (2011). Novel function of keratins 5 and 14 in proliferation and differentiation of stratified epithelial cells. Mol. Biol. Cell.

[8] Cohen E., Johnson C., Redmond C.J., Nair R.R., Coulombe P.A. (2022). Revisiting the significance of keratin expression in complex epithelia. J. Cell Sci..

[9] Callens S.J., Uyttendaele R.J., Fratila-Apachitei L.E., Zadpoor A.A. (2020). Substrate curvature as a cue to guide spatiotemporal cell and tissue organization. Biomaterials.

[10] Mierke C.T. (2024). Extracellular matrix cues regulate mechanosensing and mechanotransduction of cancer cells. Cells.

[11] Pankratova M.D., Riabinin A.A., Butova E.A., Selivanovskiy A.V., Morgun E.I., Ulianov S.V., Vorotelyak E.A., Kalabusheva E.P. (2024). YAP/TAZ Signalling Controls Epidermal Keratinocyte Fate. Int. J. Mol. Sci..

[12] Echeverria Molina M.I., Malollari K.G., Komvopoulos K. (2021). Design challenges in polymeric scaffolds for tissue engineering. Front. Bioeng. Biotechnol..

[13] Ramírez-Ruiz F., Núñez-Tapia I., Piña-Barba M.C., Alvarez-Pérez M.A., Guarino V., Serrano-Bello J. (2025). Polycaprolactone for hard tissue regeneration: Scaffold design and in vivo implications. Bioengineering.

[14] Nguyen-Truong M., Li Y.V., Wang Z. (2020). Mechanical Considerations of Electrospun Scaffolds for Myocardial Tissue and Regenerative Engineering. Bioengineering.

[15] Riaz T., Khenoussi N., Rata D.M., Atanase L.I., Adolphe D.C., Delaite C. (2023). Blend Electrospinning of Poly(Ɛ-Caprolactone) and Poly(Ethylene Glycol-400) Nanofibers Loaded with Ibuprofen as a Potential Drug Delivery System for Wound Dressings. AUTEX Res. J..

[16] Andersson A.-S., Bäckhed F., von Euler A., Richter-Dahlfors A., Sutherland D., Kasemo B. (2003). Nanoscale features influence epithelial cell morphology and cytokine production. Biomaterials.

[17] Miyoshi H., Adachi T. (2014). Topography design concept of a tissue engineering scaffold for controlling cell function and fate through actin cytoskeletal modulation. Tissue Eng. Part B Rev..

[18] Poyraz Ş., Altınışık Z., Çakmak A.S., Şimşek M., Gümüşderelioğlu M. (2022). Random/aligned electrospun PCL fibrous matrices with modified surface textures: Characterization and interactions with dermal fibroblasts and keratinocytes. Colloids Surf. B Biointerfaces.

[19] Zhou T., Wang N., Xue Y., Ding T., Liu X., Mo X., Sun J. (2016). Electrospun tilapia collagen nanofibers accelerating wound healing via inducing keratinocytes proliferation and differentiation. Colloids Surf. B Biointerfaces.

[20] Fu X., Xu M., Liu J., Qi Y., Li S., Wang H. (2014). Regulation of migratory activity of human keratinocytes by topography of multiscale collagen-containing nanofibrous matrices. Biomaterials.

[21] Pelipenko J., Kocbek P., Kristl J. (2015). Nanofiber diameter as a critical parameter affecting skin cell response. Eur. J. Pharm. Sci..

[22] Roesch-Ely M., Steinberg T., Bosch F.X., Müssig E., Whitaker N., Wiest T., Kohl A., Komposch G., Tomakidi P. (2006). Organotypic co-cultures allow for immortalized human gingival keratinocytes to reconstitute a gingival epithelial phenotype in vitro. Differentiation.

[23] Flores-Rojas G.G., Gómez-Lazaro B., López-Saucedo F., Vera-Graziano R., Bucio E., Mendizábal E. (2023). Electrospun Scaffolds for Tissue Engineering: A Review. Macromol.

[24] Gu Z., Fan S., Kundu S.C., Yao X., Zhang Y. (2023). Fiber diameters and parallel patterns: Proliferation and osteogenesis of stem cells. Regen. Biomater..

[25] Narayanan N., Jiang C., Wang C., Uzunalli G., Whittern N., Chen D., Jones O.G., Kuang S., Deng M. (2020). Harnessing Fiber Diameter-Dependent Effects of Myoblasts Toward Biomimetic Scaffold-Based Skeletal Muscle Regeneration. Front. Bioeng. Biotechnol..

[26] Robinson A.J., Pérez-Nava A., Ali S.C., González-Campos J.B., Holloway J.L., Cosgriff-Hernandez E.M. (2021). Comparative Analysis of Fiber Alignment Methods in Electrospinning. Matter.

[27] Selig M., Azizi S., Walz K., Lauer J.C., Rolauffs B., Hart M.L. (2023). Cell morphology as a biological fingerprint of chondrocyte phenotype in control and inflammatory conditions. Front. Immunol..

[28] Selig M., Poehlman L., Lang N.C., Völker M., Rolauffs B., Hart M.L. (2024). Prediction of six macrophage phenotypes and their IL-10 content based on single-cell morphology using artificial intelligence. Front. Immunol..

[29] Livak K.J., Schmittgen T.D. (2001). Analysis of relative gene expression data using real-time quantitative PCR and the 2− ΔΔCT method. Methods.

[30] Ramminger I. (2025). Influence of Biophysical Cues on Gingival Keratinocyte Behavior: Exploring Fiber Orientation and Diameter for Next-Generation Oral Epithelial Regeneration. Ph.D. Thesis.

[31] Xue J., Wu T., Xia Y. (2018). Perspective: Aligned arrays of electrospun nanofibers for directing cell migration. APL Mater..

[32] Lobo J., See E.Y.-S., Biggs M., Pandit A. (2016). An insight into morphometric descriptors of cell shape that pertain to regenerative medicine. J. Tissue Eng. Regen. Med..

[33] Streichan S.J., Hoerner C.R., Schneidt T., Holzer D., Hufnagel L. (2014). Spatial constraints control cell proliferation in tissues. Proc. Natl. Acad. Sci. USA.

[34] Nathan A.S., Baker B.M., Nerurkar N.L., Mauck R.L. (2011). Mechano-topographic modulation of stem cell nuclear shape on nanofibrous scaffolds. Acta Biomater..

[35] Kim J.Y., Quan T. (2024). Emerging perspectives of YAP/TAZ in human skin epidermal and dermal aging. Ann. Dermatol..

[36] Wang A.S., Ong P.F., Chojnowski A., Clavel C., Dreesen O. (2017). Loss of lamin B1 is a biomarker to quantify cellular senescence in photoaged skin. Sci. Rep..

[37] Klein C., Ramminger I., Bai S., Steinberg T., Tomakidi P. (2024). Impairment of Intermediate Filament Expression Reveals Impact on Cell Functions Independent from Keratinocyte Transformation. Cells.

[38] Bhattacharjee P., Cavanagh B.L., Ahearne M. (2020). Effect of substrate topography on the regulation of human corneal stromal cells. Colloids Surf. B Biointerfaces.

[39] Teixeira A.I., Abrams G.A., Bertics P.J., Murphy C.J., Nealey P.F. (2003). Epithelial contact guidance on well-defined micro-and nanostructured substrates. J. Cell Sci..

[40] Putra V.D., Kilian K.A., Knothe Tate M.L. (2023). Biomechanical, biophysical and biochemical modulators of cytoskeletal remodelling and emergent stem cell lineage commitment. Commun. Biol..

[41] Zhou W., Lin J., Wang Q., Wang X., Yao X., Yan Y., Sun W., Zhu Q., Zhang X., Wang X. (2025). Chromatin-site-specific accessibility: A microtopography-regulated door into the stem cell fate. Cell Rep..

[42] Paul C.D., Hruska A., Staunton J.R., Burr H.A., Daly K.M., Kim J., Jiang N., Tanner K. (2019). Probing cellular response to topography in three dimensions. Biomaterials.

[43] Sakamoto R., Murrell M.P. (2024). Substrate geometry and topography induce F-actin reorganization and chiral alignment in an adherent model cortex. Cell Rep. Phys. Sci..

[44] Carthew J., Abdelmaksoud H.H., Hodgson-Garms M., Aslanoglou S., Ghavamian S., Elnathan R., Spatz J.P., Brugger J., Thissen H., Voelcker N.H. (2021). Precision surface microtopography regulates cell fate via changes to actomyosin contractility and nuclear architecture. Adv. Sci..

[45] Hoffman L.M., Smith M.A., Jensen C.C., Yoshigi M., Blankman E., Ullman K.S., Beckerle M.C. (2020). Mechanical stress triggers nuclear remodeling and the formation of transmembrane actin nuclear lines with associated nuclear pore complexes. Mol. Biol. Cell.

[46] Law J.X., Liau L.L., Saim A., Yang Y., Idrus R. (2017). Electrospun collagen nanofibers and their applications in skin tissue engineering. Tissue Eng. Regen. Med..

[47] Chi J., Wang M., Chen J., Hu L., Chen Z., Backman L.J., Zhang W. (2022). Topographic orientation of scaffolds for tissue regeneration: Recent advances in biomaterial design and applications. Biomimetics.

[48] Coşkun G., Karaca E., Ozyurtlu M., Özbek S., Yermezler A., Çavuşoğlu İ. (2014). Histological evaluation of wound healing performance of electrospun poly (vinyl alcohol)/sodium alginate as wound dressing in vivo. Bio-Med. Mater. Eng..

[49] Belgardt E., Steinberg T., Husari A., Dieterle M.P., Hülter-Hassler D., Jung B., Tomakidi P. (2020). Force-responsive Zyxin modulation in periodontal ligament cells is regulated by YAP rather than TAZ. Cell. Signal..

[50] Hülter-Hassler D., Wein M., Schulz S.D., Proksch S., Steinberg T., Jung B.A., Tomakidi P. (2017). Biomechanical strain-induced modulation of proliferation coincides with an ERK1/2-independent nuclear YAP localization. Exp. Cell Res..

[51] Bermudez-Lekerika P., Crump K.B., Wuertz-Kozak K., Le Maitre C.L., Gantenbein B. (2024). Sulfated hydrogels as primary intervertebral disc cell culture systems. Gels.

[52] Careta O., Salicio-Paz A., Pellicer E., Ibáñez E., Fornell J., García-Lecina E., Sort J., Nogués C. (2021). Electroless palladium-coated polymer scaffolds for electrical stimulation of osteoblast-like saos-2 cells. Int. J. Mol. Sci..

[53] Sindhi K., Pingili R.B., Beldar V., Bhattacharya S., Rahaman J., Mukherjee D. (2025). The role of biomaterials-based scaffolds in advancing skin tissue construct. J. Tissue Viability.

[54] Lestari W., Irfanita N., Haris M.S., Lin G.S.S., Jaswir I., Darnis D.S., Ruziantee N., Mazlan N., Idrus E., Amir L.R. (2026). Advancements and applications of gelatin-based scaffolds in dental engineering: A narrative review. Odontology.

[55] Cheng B., Li M., Wan W., Guo H., Genin G.M., Lin M., Xu F. (2023). Predicting YAP/TAZ nuclear translocation in response to ECM mechanosensing. Biophys. J..

[56] Jedrusik N., Meyen C., Finkenzeller G., Stark G.B., Meskath S., Schulz S.D., Steinberg T., Eberwein P., Strassburg S., Tomakidi P. (2018). Nanofibered gelatin-based nonwoven elasticity promotes epithelial Histogenesis. Adv. Healthc. Mater..

