# Supplementary material for: Form Meets Function: Fiber Architecture Directs Proliferation and Differentiation in Gingival Keratinocytes
Source: Cells. 2026 Feb 5;15(3):300. doi: 10.3390/cells15030300 (PMC12896891; doi:10.3390/cells15030300)
Supplement: Supplementary file 1 [file cells-15-00300-s001.zip › Supplemental Information MDPI Cells_TS_V3_IR (002).pdf]

Supplemental Material:

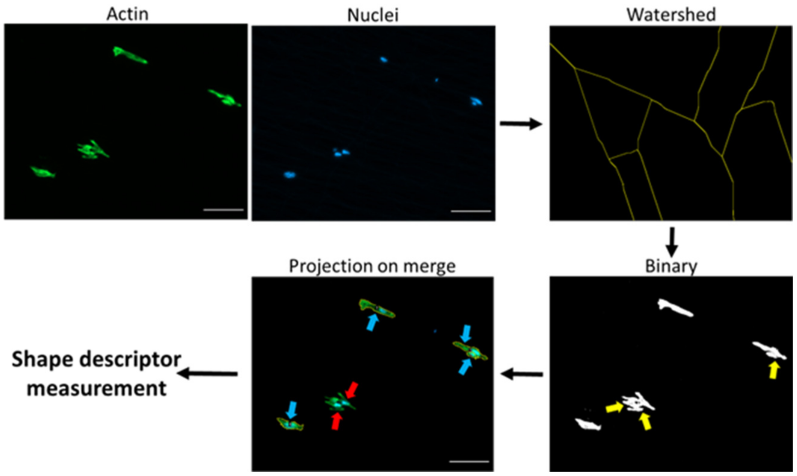

Figure S1. ImageJ/Fiji-based workflow for quantitative analysis of cell morphology in ihGK cells cultured on aligned scaffolds. Representative images depict ihGK cells cultured for three days on 1.2–1.7 µm aligned scaffolds. Cells were stained for F-actin (green) and nuclei (blue), and imaged at 20× magnification. Nuclei were segmented using the Watershed algorithm, with separation lines shown in yellow. A binary mask was generated to identify individual cell particles, with examples of watershed separation indicated by yellow arrows. Particle contours were subsequently projected onto the merged fluorescence image. Correctly segmented cells are marked with blue arrows, while red arrows denote segmentation errors. Only correctly segmented cells were included in the quantitative shape descriptor analysis. Scale bars: 100 µm. [1].

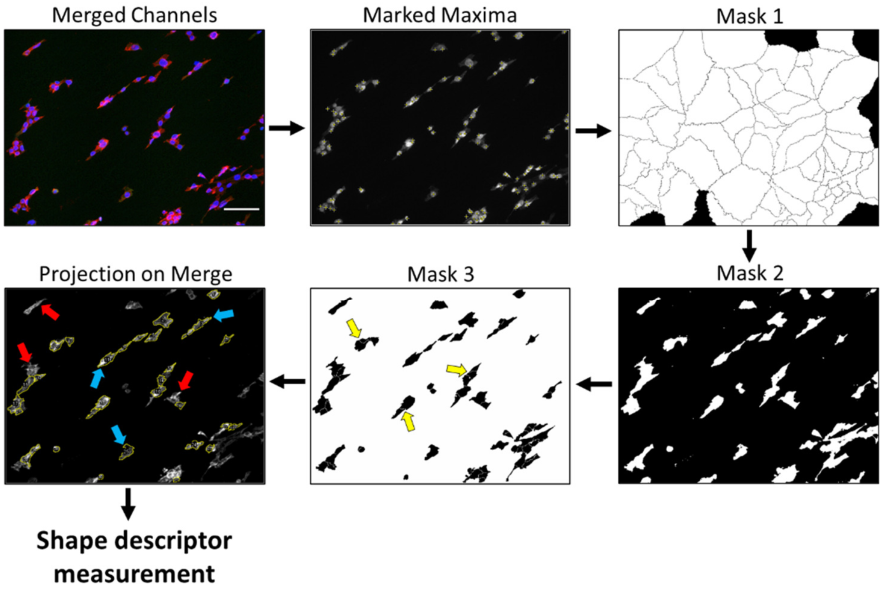

Figure S2. Semi-automated Fiji/ImageJ-based workflow for morphological analysis of ihGK cells cultured on 600–800 nm aligned scaffolds (batch 2) for three days. Cells were stained for nuclei (blue), actin (red), and KRT14 (green; weak signal), and imaged at 20× magnification. Cell morphology was analyzed using a semi-automated Fiji/ImageJ pipeline. The Find Maxima function was applied to detect local intensity peaks, resulting in a segmentation output (Mask 1). Background was excluded using thresholding (Mask 2). Overlaying Masks 1 and 2 generated a refined binary mask (Mask 3), enabling the visualization of segmented cell boundaries; separation lines are indicated by yellow arrows. Segmented contours were projected onto the merged fluorescence image to

facilitate classification of correctly segmented cells (blue arrows) and segmentation errors (red arrows). Only correctly identified cells were included in downstream shape descriptor analysis. Scale bar: 100 µm. [1].

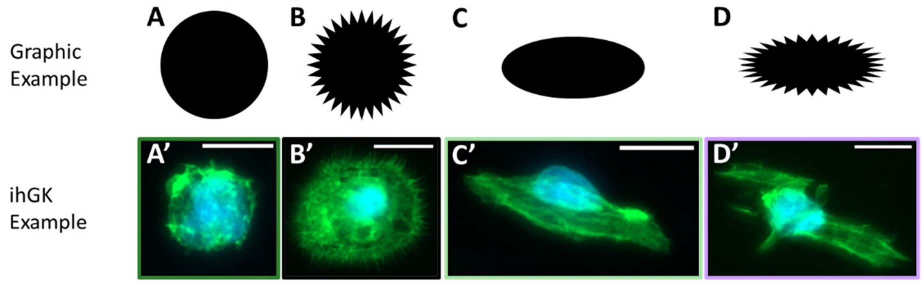

Area [µm²]	569.456	1451.909	740.1995	905.7362
Aspect ratio	1.0187	1.2066	3.4535	3.1227
Roundness	0.9817	0.8288	0.2896	0.3202
Circularity	0.4787	0.0808	0.3451	0.1218
Solidity	0.9053	0.8432	0.8874	0.5900

Figure S3. Quantitative shape descriptors used for morphometric analysis of ihGK cells. Schematic representations (A–D) illustrate idealized geometric profiles used to conceptualize shape descriptors. Corresponding fluorescence microscopy images (A'–D') depict representative ihGK cells cultured on different scaffold types: (A') 600–800 nm random, (B') flat control surface, (C') 600–800 nm aligned scaffold, and (D') 1.2–1.7 µm aligned scaffold. All samples represent cells from batch 1. Cells were stained for F-actin (green) and nuclei (blue), and imaged using a 20× objective. The table summarizes measured shape descriptors for each cell, including area, aspect ratio, roundness, circularity, and solidity. These metrics were extracted using Fiji/ImageJ and illustrate the morphological variability induced by substrate architecture. Images show cropped regions of the full field of view. Scale bars: 20 µm. [1].

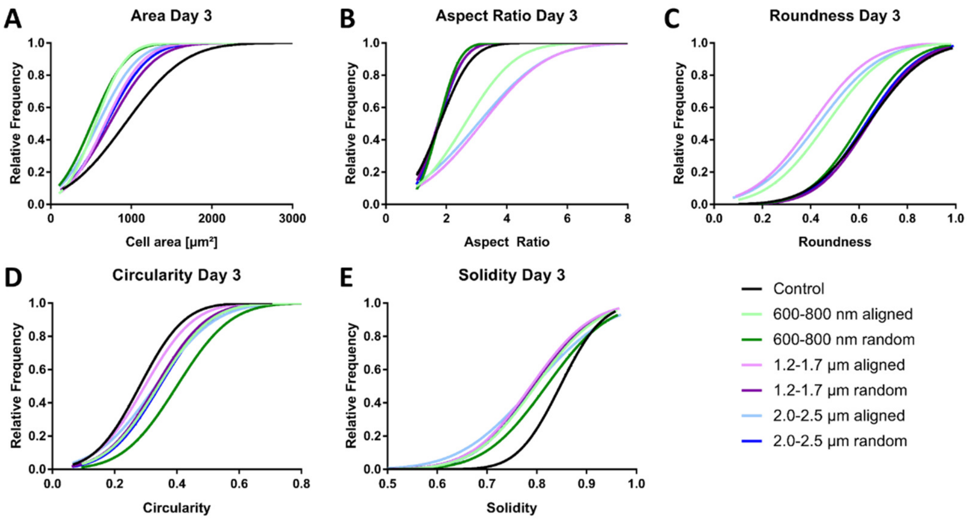

Figure S4. Cumulative distribution plots of ihGK cell shape descriptors after three days of culture on different scaffold topographies (batch 1). Morphometric analysis of ihGK cells cultured for three days on substrates with varying surface architectures. Cumulative distribution curves are shown for five distinct shape descriptors: (A) cell area (µm²), (B) aspect ratio, (C) roundness, (D) circularity, and (E) solidity. Cell shape quantification was performed using a semi-automated Fiji/ImageJ-based image analysis workflow. Data represent four independent biological replicates per condition (n=4). Cell counts per group were as follows: 600–800 nm aligned (n = 1114), 600–800 nm random (n = 1138), 1.2–1.7 µm aligned (n = 644), 1.2–1.7 µm random (n = 625), 2.0–2.5 µm aligned (n = 585), 2.0–2.5 µm random (n = 678), and control (n = 675). [1].

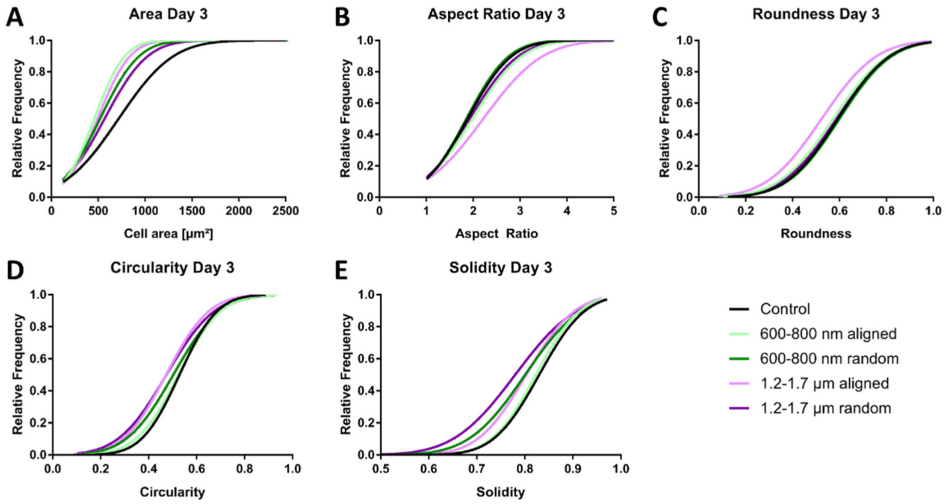

Figure S5. Cumulative distribution analysis of ihGK cell shape descriptors after three days of culture on different scaffold types (batch 2). Cumulative distribution plots illustrating morphometric parameters of ihGK cells cultured on substrates with distinct nanotopographies for three days. Parameters analyzed include (A) cell area (μm²), (B) aspect ratio, (C) roundness, (D) circularity, and (E) solidity. Quantification was performed using a semi-automated Fiji/ImageJ-based analysis workflow. Data were obtained from three biological replicates (n=3). Exact cell counts were: 600–800 nm aligned (n = 4692), 600–800 nm random (n = 3298), 1.2–1.7 μm aligned (n = 4624), 1.2–1.7 μm random (n = 2478), and control (n = 3471). [1].

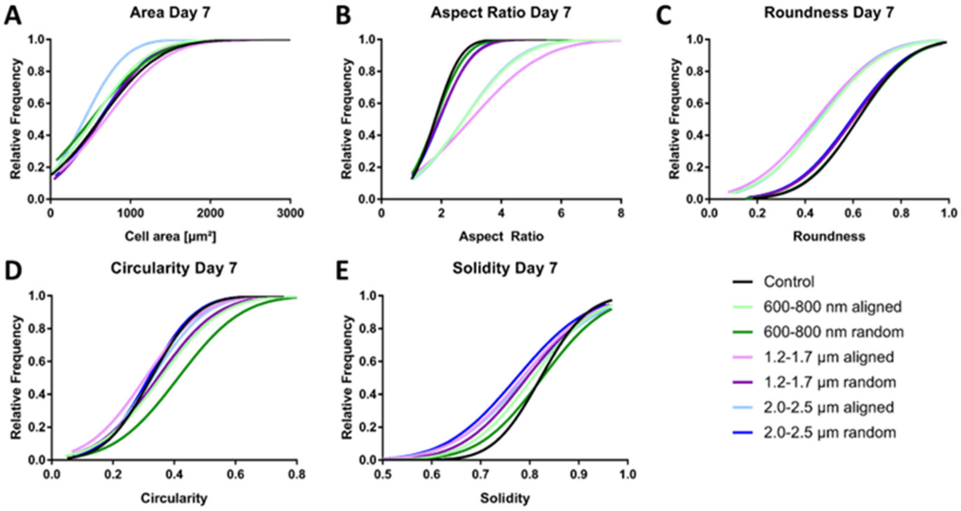

Figure S6. Cumulative distribution plots of ihGK cell shape descriptors after seven days of culture on different scaffold topographies (batch 1). Cumulative distribution curves illustrating morphometric characteristics of ihGK cells after seven days of cultivation on various surface structures. Parameters analyzed include (A) cell area (μm²), (B) aspect ratio, (C) roundness, (D) circularity, and (E) solidity. Quantitative assessment was carried out using a semi-automated Fiji/ImageJ-based image analysis approach. Data represent four biological replicates (n=4). Exact cell counts per group: 600–800 nm aligned (n = 422), 600–800 nm random (n = 352), 1.2–1.7 μm aligned (n = 235), 1.2–1.7 μm random (n = 201), 2.0–2.5 μm aligned (n = 411), 2.0–2.5 μm random (n = 178), and control (n = 788). [1].

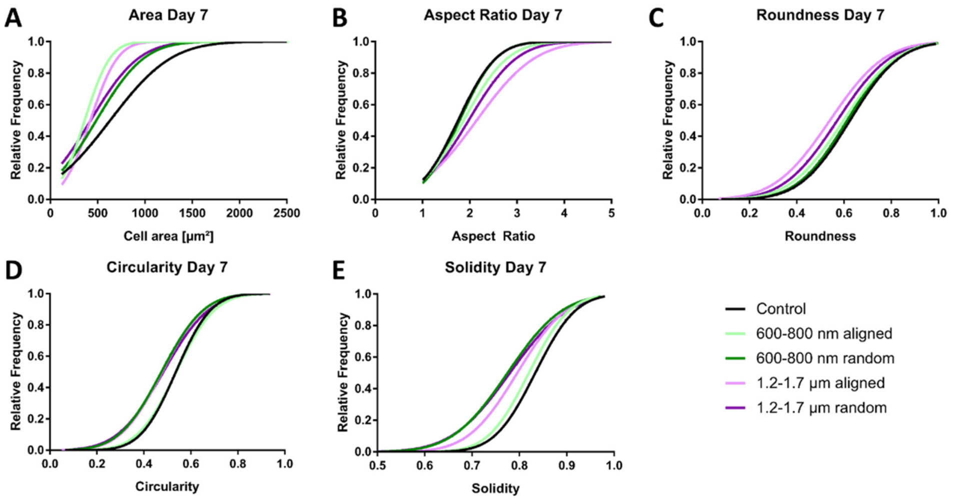

Figure S7. Cumulative distribution analysis of ihGK cell shape descriptors after seven days of culture on different scaffold architectures (batch 2). Cumulative distribution plots display morphometric parameters of ihGK cells after seven days of cultivation on various substrate topographies. The measured shape descriptors include (A) cell area (µm²), (B) aspect ratio, (C) roundness, (D) circularity, and (E) solidity. Cell morphology was quantified using a semi-automated Fiji/ImageJ-based image analysis workflow. Data represent three independent biological replicates (n=3). Exact cell numbers were as follows: 600–800 nm aligned (n = 9433), 600–800 nm random (n = 7207), 1.2–1.7 µm aligned (n = 11,355), 1.2–1.7 µm random (n = 4735), and control (n = 16,603). [1].

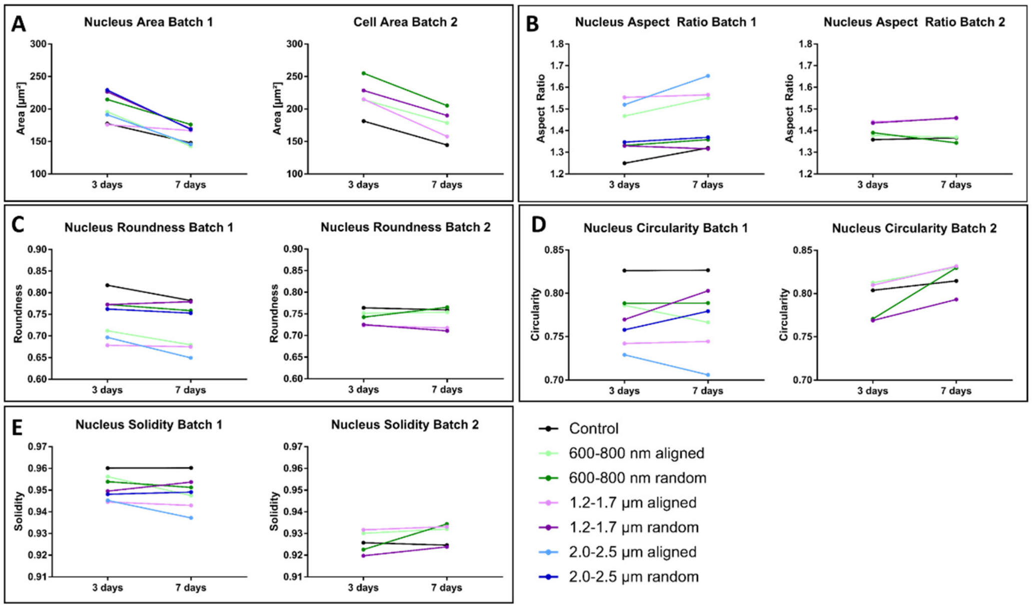

Figure S8. Mean values of nuclear shape descriptors of ihGK cells cultured on different scaffold topographies at day 3 and day 7 for batch 1 and batch 2. Line plots depict the temporal changes in mean nuclear morphology parameters of ihGK cells cultured on varying surface architectures at day 3 and day 7. Data are shown separately for batch 1 (left panels, n = 4 biological replicates) and batch 2 (right panels, n = 3 biological replicates). Quantified nuclear shape descriptors include: (A) nucleus area (µm²), (B) aspect ratio, (C) roundness, (D) circularity, and (E)

solidity. Mean values were calculated from all biological replicates within each batch. Corresponding standard deviation (SD) values are provided in Table 3 (day 3) and Table 4 (day 7). [1].

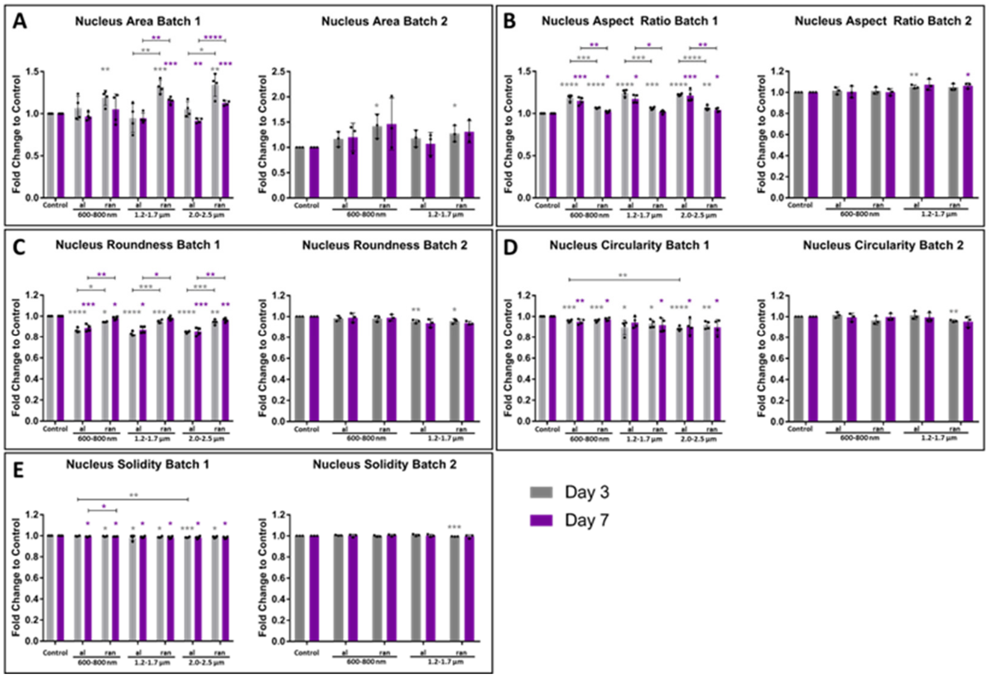

Figure S9. Temporal changes in nuclear shape descriptors of ihGK cells on different scaffold architectures, normalized to control conditions at day 3 and day 7. Bar plots display the fold change in mean values of nuclear shape descriptors in ihGK cells cultured on nanotopographic scaffolds, normalized to corresponding values from cells cultured on flat control surfaces. Parameters analyzed include: (A) nucleus area, (B) aspect ratio, (C) roundness, (D) circularity, and (E) solidity. Fold changes were calculated for each biological replicate individually, resulting in n = 4 for batch 1 and n = 3 for batch 2. Data normality was assessed using the Shapiro–Wilk test. Statistical comparisons were performed using unpaired two-tailed t-tests for normally distributed data or Mann–Whitney tests for non-normally distributed data. Asterisks above individual bars indicate statistical significance versus the control group. Error bars represent standard deviation (SD). Significance thresholds: $p < 0.05$ (*), $p < 0.01$ (**), $p < 0.001$ (***), $p < 0.0001$ (****). Abbreviations: al = aligned, ran = random. [1].

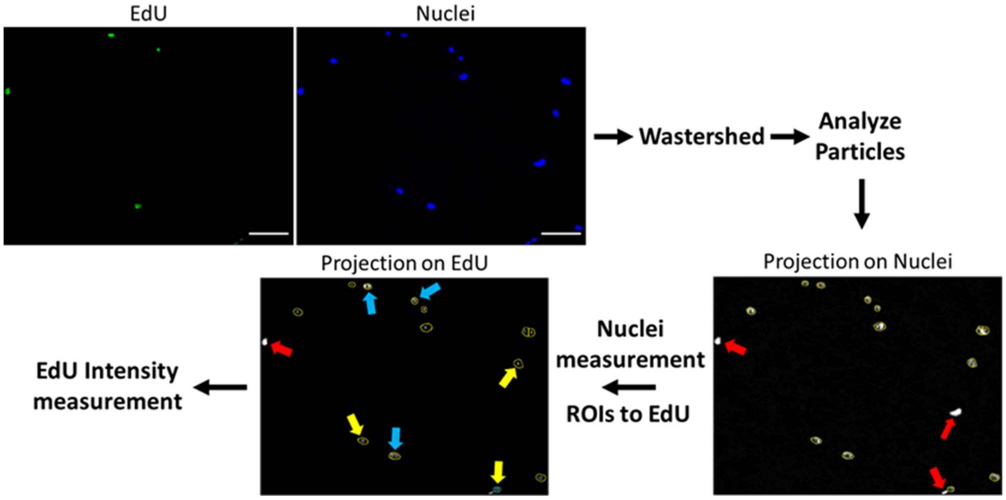

Figure S10. Fiji/ImageJ-based image analysis workflow for quantifying proliferation activity in ihGK cells using EdU incorporation. Workflow for the semi-automated detection and quantification of proliferating ihGK cells based on EdU incorporation. Cells were stained for EdU (green), marking cells undergoing DNA synthesis, and

for nuclei (blue). Following nuclei segmentation using the Watershed function, nuclear regions were identified via the Analyze Particles tool in Fiji/ImageJ. The identified regions of interest (ROIs) corresponding to nuclei were saved and projected back onto both the nuclei and EdU channels for verification and intensity analysis. Red arrows indicate nuclei not recognized by the segmentation algorithm. EdU intensity was measured within the nuclear ROIs, and proliferating cells (blue arrows) were distinguished from non-proliferating cells (yellow arrows) based on an established intensity threshold. Scale bar: 100 μm. [1].

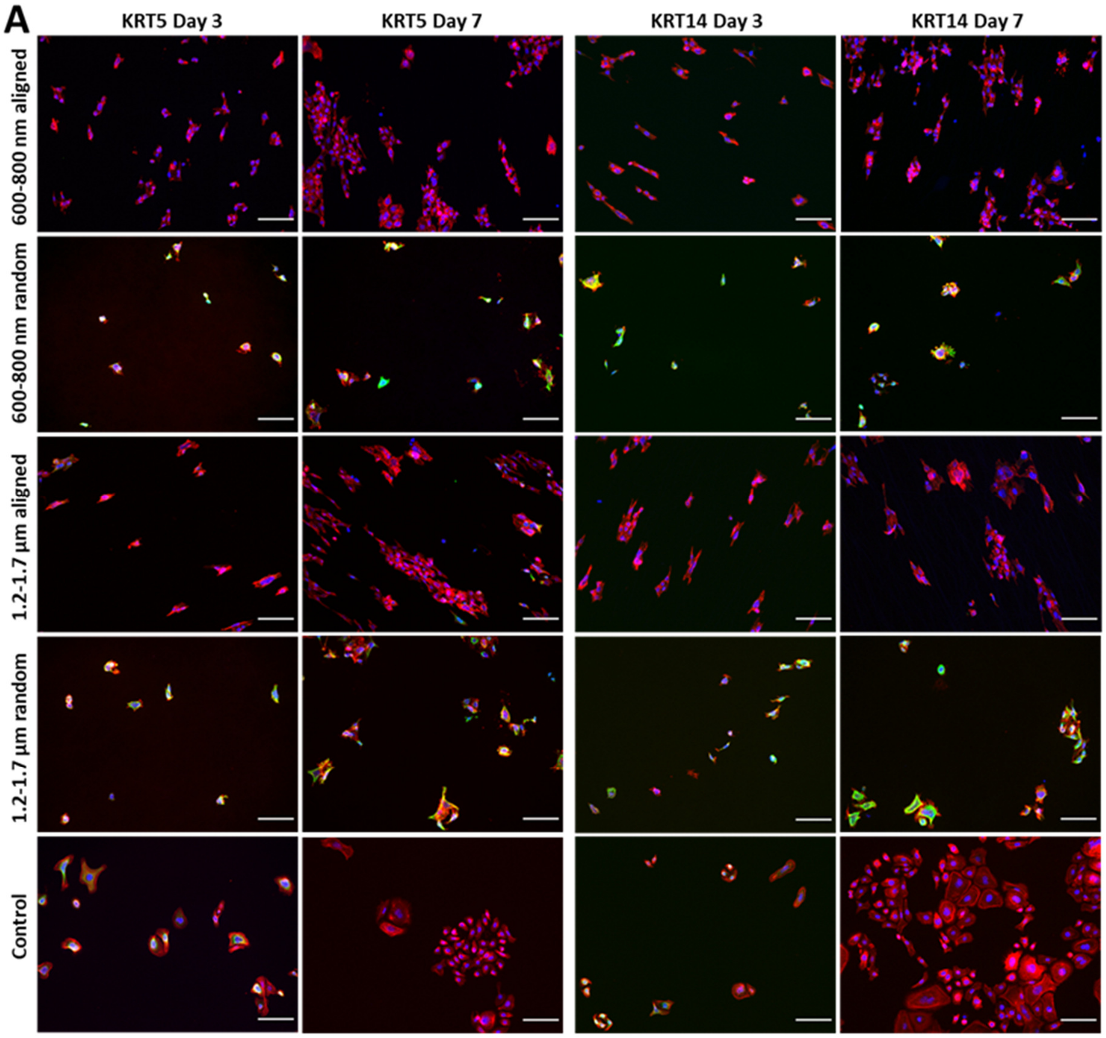

Figure S11. Basal cell marker expression in ihGK cells cultured on different scaffold surfaces after 3 and 7 days. Representative fluorescence microscopy images of ihGK cells stained for actin (red), nuclei (blue), and the basal cell markers KRT5 or KRT14 (green), acquired after 3 or 7 days of culture on various surface topographies. Exposure time for protein imaging was kept constant across all samples. Images were captured using a 20× objective. Scale bar: 100 μm. [1].

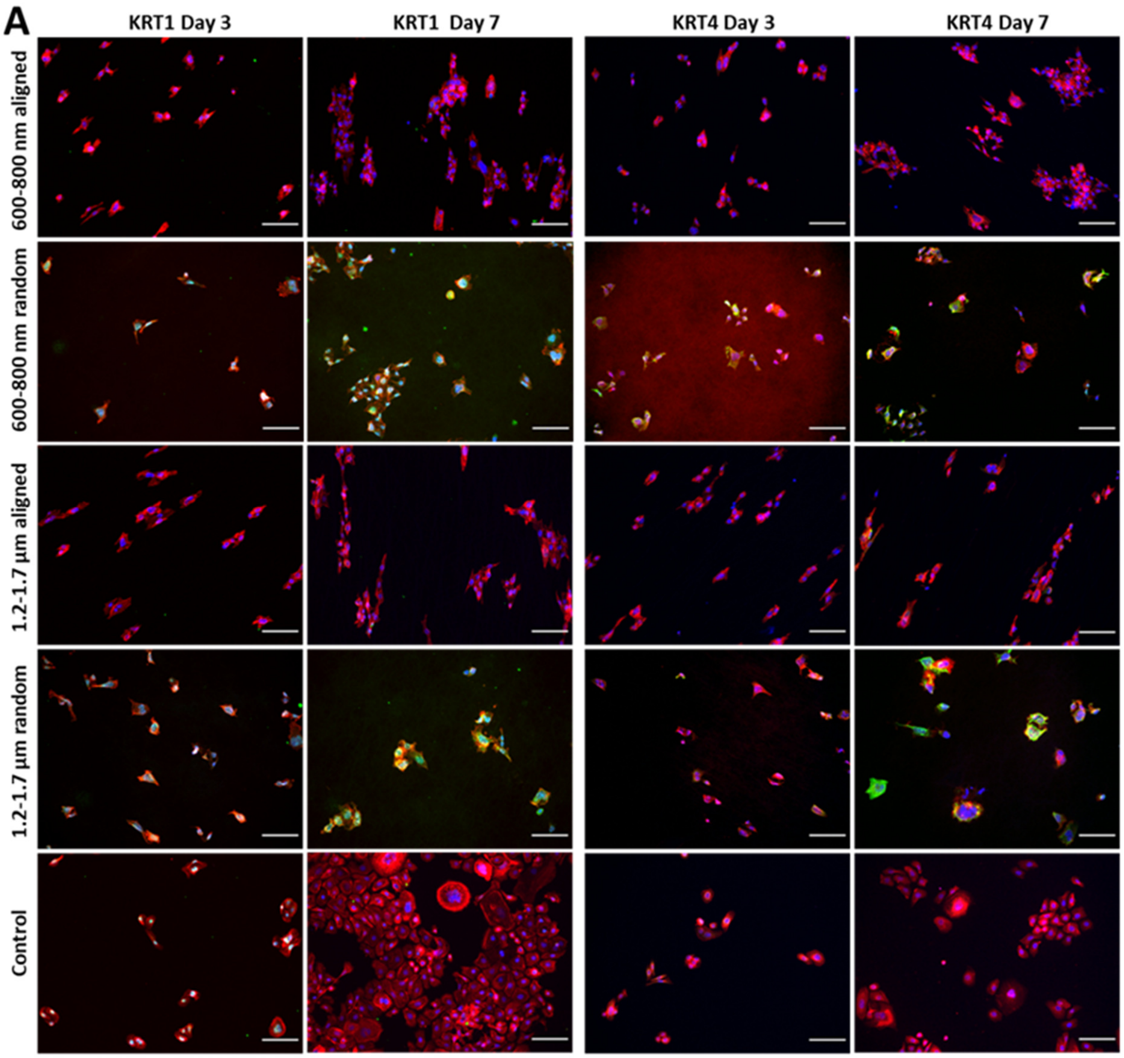

Figure S12. Expression of early differentiation markers KRT1 and KRT4 in ihGK cells cultured on different scaffold surfaces after 3 and 7 days. Representative immunofluorescence images of ihGK cells stained for actin (red), nuclei (blue), and the early differentiation markers KRT1 or KRT4 (green). Images were acquired at 20× magnification under identical exposure settings across all conditions. Scale bar: 100 µm. [1].

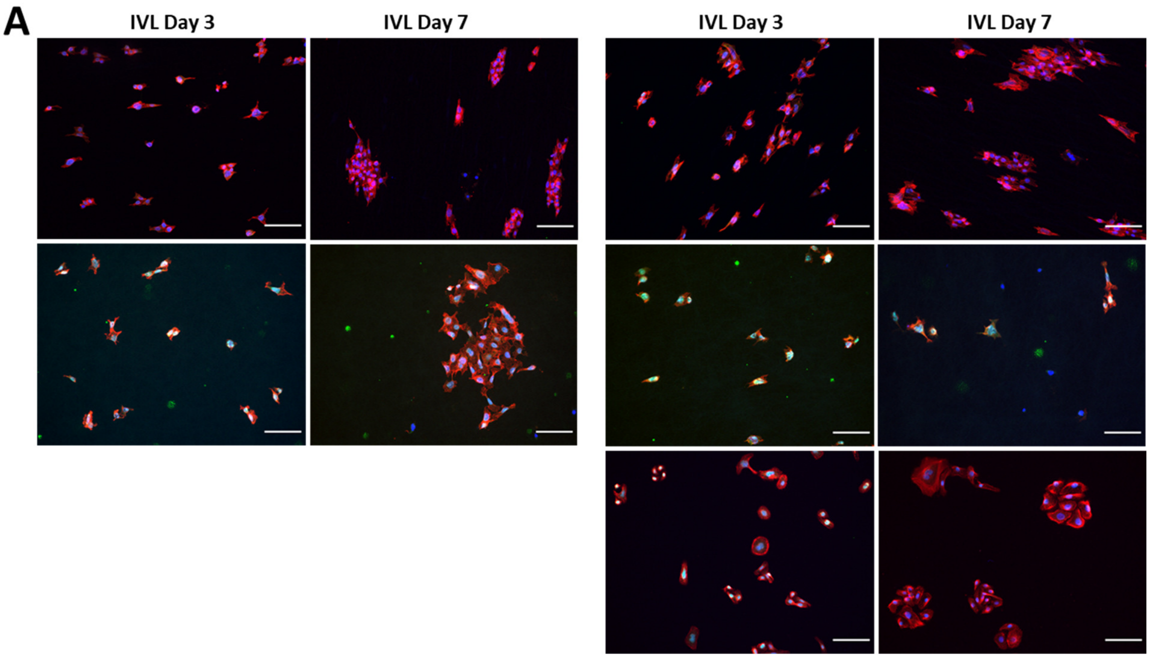

Figure S13. Expression of the terminal differentiation marker IVL in ihGK cells cultured on different scaffold surfaces after 3 and 7 days. Representative immunofluorescence images of ihGK cells stained for actin (red), nuclei (blue), and involucrin (IVL, green), acquired after 3 and 7 days of culture on various surface topographies. Protein exposure settings were kept constant across all conditions. Images were taken using a 20× objective. Scale bar: 100 μm. [1].

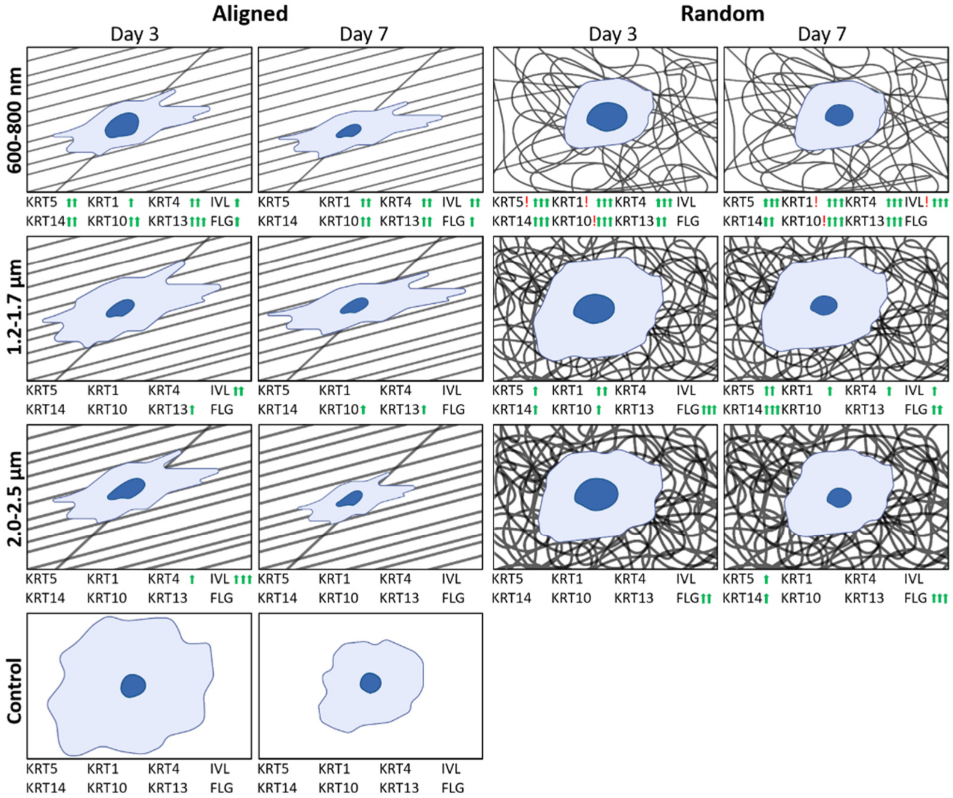

Figure S14. Schematic illustration of ihGK cell responses to different scaffold topographies after 3 and 7 days of culture (batch 1). Schematic overviews depict ihGK cell morphology, organization, and differentiation responses on various scaffold types following 3 and 7 days of cultivation in batch 1. The illustrations were created using

BioRender.com and are based on experimental findings from morphological, and gene expression analyses. They are conceptual representations intended to summarize the overall biological trends rather than depict exact morphologies or scales. Surface architecture is reflected in the scaffold designs, with notable differences between fiber arrangements: the 600–800 nm random condition displays a distinct weaving pattern compared to the 1.2–1.7 µm and 2.0–2.5 µm random scaffolds, which exhibit more linear fiber alignments. RNA expression levels of key genes were ranked and visualized using a symbolic arrow system beneath each scaffold condition:

- ↑↑↑ = highest expression,
- ↑↑ = third highest,
- ↑ = fourth highest.

A red exclamation mark (!) indicates a particularly elevated expression level of the corresponding gene.

No scale is provided for the illustrations. [1].

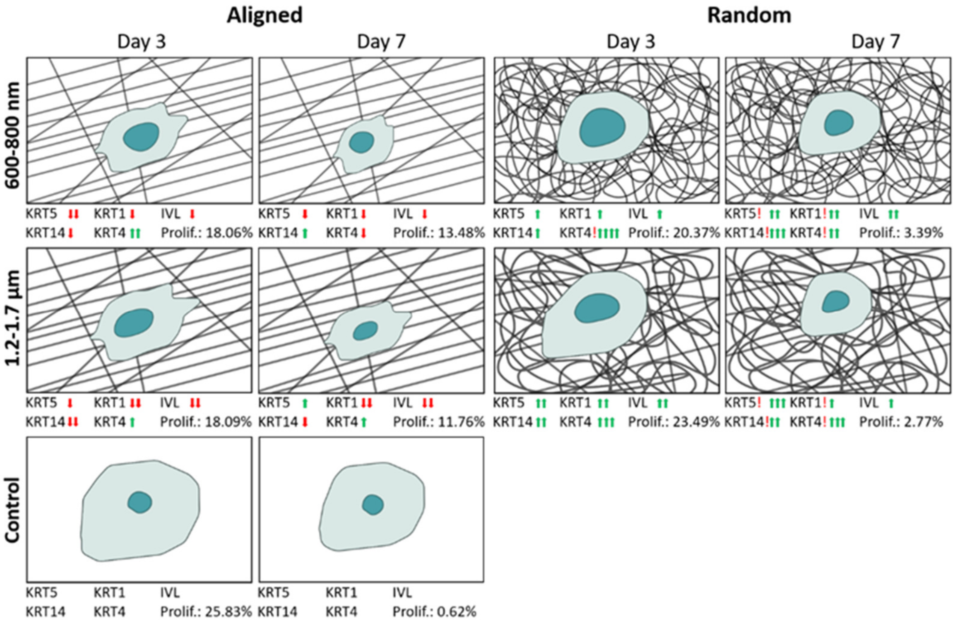

Figure S15. Schematic overview of ihGK cell behavior on various scaffold surfaces after 3 and 7 days of culture (batch 2). This graphical summary illustrates ihGK cell responses to different nanotopographic scaffolds at day 3 and day 7 of cultivation in batch 2. The schematics were generated using BioRender.com and are based on quantitative and qualitative measurements, including protein expression, proliferation, and scaffold architecture. They serve as visual representations to highlight trends in cell behavior rather than scaled or anatomically accurate images. Scaffold representations reflect key structural features observed across conditions. Notably, the 600–800 nm random scaffolds displayed a distinct weaving pattern, while the 1.2–1.7 µm random scaffolds exhibited more linear fiber alignment. Compared to batch 1, aligned scaffolds in batch 2 demonstrated increased fiber cross-linking. Protein expression levels were ranked relative to control cells and visualized using a green and red arrow system:

↑↑↑↑ = highest expression,

↑↑↑ = second highest,

↑↑ = third highest,

↑ = fourth highest.

Reduced expression compared to control is indicated by red arrows:

↓↓ = lowest,

↓

= second lowest.

A red exclamation mark (!) indicates exceptionally elevated expression of a given protein marker. Proliferation is shown as a percentage of EdU-positive cells relative to the total number of nuclei. No scale is provided for these illustrations. Abbreviation: Prolif. = Proliferation; al = aligned; ran = random. [1].

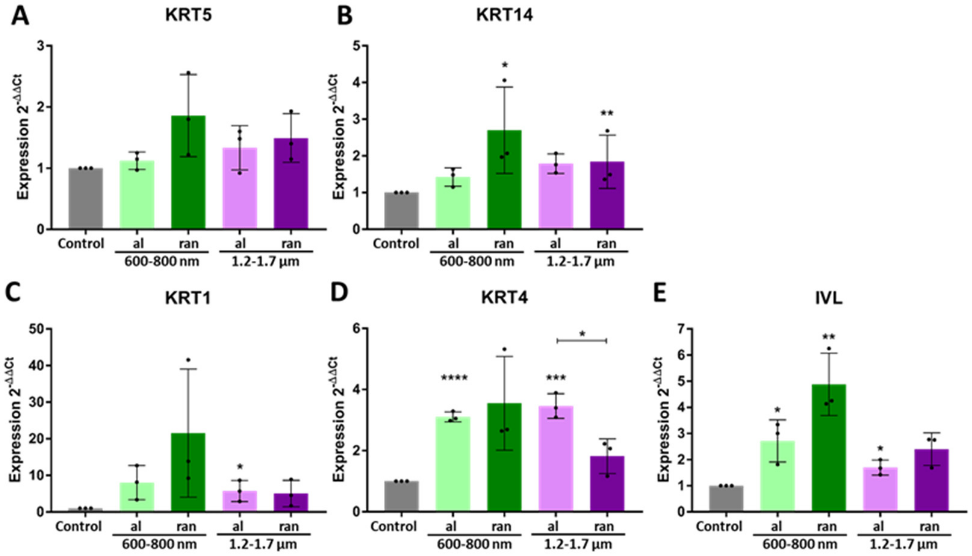

Figure S16. Expression of differentiation markers in ihGK cells cultured for 96 h without treatment on various scaffold surfaces (batch 2). ihGK cells were seeded at higher density and cultured for 96 hours under standard, untreated conditions on different nanotopographic surfaces (batch 2). Total RNA was extracted and analyzed by quantitative PCR (qPCR) for the expression of key differentiation markers. Relative expression levels were calculated in comparison to cells cultured on flat control surfaces. Gene targets included: (A) KRT5 and (B) KRT14 (basal markers), (C) KRT1 and (D) KRT4 (early differentiation markers), (E) IVL (terminal differentiation marker). Each data point represents a biological replicate (n = 3). Error bars indicate standard deviation (SD). Normality of data distribution was assessed using the Shapiro–Wilk test. Unpaired two-tailed t-tests were applied to normally distributed data, while non-normally distributed data were analyzed using the Mann–Whitney test. Statistical significance compared to the control is indicated by asterisks above the bars. $p < 0.05$ (*), $p < 0.01$ (**), $p < 0.001$ (***), $p < 0.0001$ (****). Abbreviations: al = aligned, ran = random. [1].

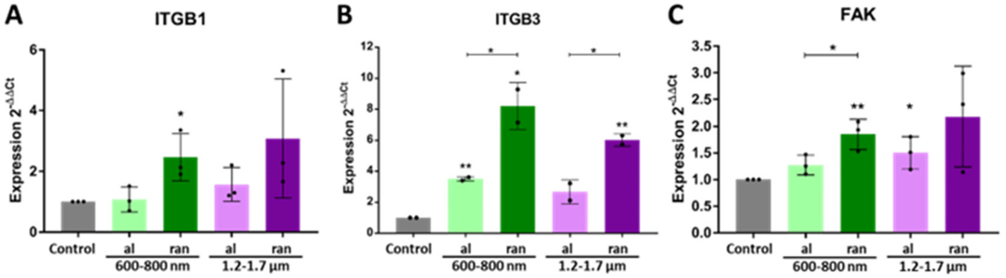

Figure S17. Expression of adhesion-related markers in ihGK cells cultured for 96 h without treatment on various scaffold surfaces (batch 2). Quantitative PCR (qPCR) analysis of adhesion marker gene expression in ihGK cells cultured for 96 hours on different nanotopographic substrates without additional treatment. Relative mRNA expression levels were determined for (A) ITGB1, (B) ITGB3, and (C) FAK, normalized to the control condition (flat surface). Each data point represents a biological replicate. Sample sizes: n = 3 for ITGB1 and FAK; n = 2 for ITGB3. Error bars indicate standard deviation (SD). Data normality was evaluated using the Shapiro–Wilk test. Statistical comparisons were performed using unpaired two-tailed t-tests for normally distributed data and

Mann–Whitney tests for non-normally distributed data. Statistical significance relative to the control is indicated by asterisks. $p < 0.05$ (*), $p < 0.01$ (**). Abbreviations: al = aligned, ran = random.[1].

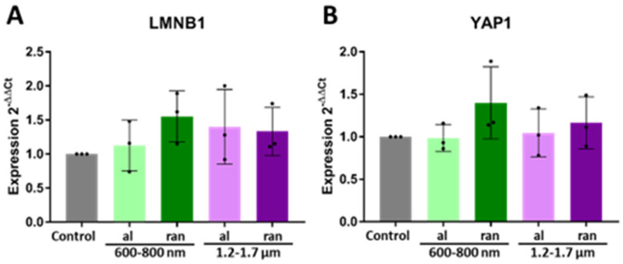

Figure S18. Gene expression of LMNB1 and YAP1 in ihGK cells after 96 hours of culture on different scaffold surfaces (batch 2). Quantitative PCR analysis of (A) LMNB1, encoding the nuclear intermediate filament lamin B1, and (B) YAP1, a co-transcriptional regulator associated with proliferation, in ihGK cells cultured for 96 hours on various nanotopographic scaffolds without additional treatment. Relative expression levels were calculated in comparison to cells cultured on flat control surfaces. No statistically significant differences in expression were observed across conditions. Data represent three biological replicates (n = 3). Abbreviations: al = aligned, ran = random.[1].

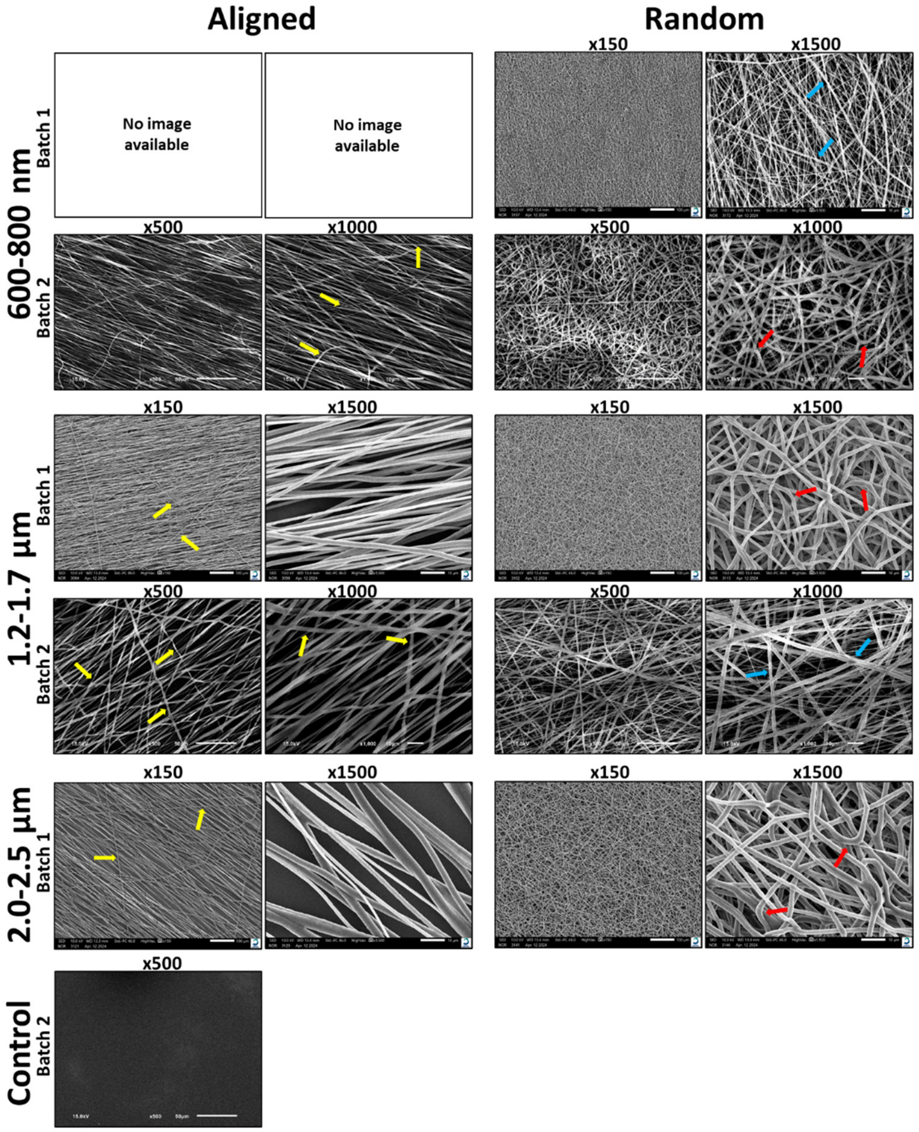

Figure S19. Scanning electron microscopy (SEM) analysis of scaffold and control surfaces without ihGK cells. SEM micrographs show the surface architecture of various scaffold conditions and the glass control surface at two magnifications. Samples were sputter-coated with a thin gold layer prior to imaging. The micrographs highlight key morphological features and differences in fiber arrangement between scaffold batches. Cross-linked fiber structures in aligned scaffolds are indicated by yellow arrows. Blue arrows mark straight lined fiber orientations for random scaffolds, whereas red arrows indicate curved fiber patterns found in random configurations. SEM images from scaffold batch 1 were provided by the research group of Prof. Dr. Bodo Kurz. No SEM image was available for the 600–800 nm aligned condition from batch 1. The control surface represents an uncoated glass coverslip used in microscopy experiments. Scale bars: ×150 = 100 μm, ×500 = 50 μm, ×1000 and ×1500 = 10 μm. n = 1 per condition. [1].

		**batch 1**	**batch 2**
**600-800 nm**	**aligned**	726 ± 198 nm	926 ± 366 nm
	**random**	716 ± 172 nm	710 ± 243 nm
**1.2-1.7 μm**	**aligned**	1.64 ± 0.51 μm	1.31 ± 0.45 μm
	**random**	1.58 ± 0.41 μm	1.63 ± 0.26 μm
**2.0-2.5 μm**	**aligned**	2.19 ± 0.78 μm	X
	**random**	2.16 ± 0.51 μm	X

Table S1. Scaffold diameter means ± SD of batch 1 and 2 scaffolds. Diameter measurements were performed from the Electrospinning® Company and specified in the provided evaluation report with scaffold delivery. [1].

References

1. Ramminger, I. Influence of Biophysical Cues on Gingival Keratinocyte Behavior: Exploring Fiber Orientation and Diameter for Next-Generation Oral Epithelial Regeneration. Ph.D. Thesis, University of Freiburg, Freiburg, Germany, 2025.
